# Improving the Quality of Low-Salt Beef Myofibrillar Protein Gels with L-Lysine and Konjac Glucomannan: Water Retention, Texture, and Protein Structural Changes

**DOI:** 10.3390/gels12080709

**Published:** 2026-08-10

**Authors:** Xiuyun Guo, Jinsheng Yang, Chao Fu, Jiangpeng Yao, Zhikun Yang, Xiangren Meng

**Affiliations:** 1 School of Tourism and Cuisine, Yangzhou University, Yangzhou 225127, China; 17551657508@163.com (J.Y.); f1610210342@gmail.com (C.F.); 15189433171@163.com (J.Y.); yangzhikun@yzu.edu.cn (Z.Y.); 2 Key Laboratory of Chinese Cuisine Intangible Cultural Heritage Technology Inheritance, Ministry of Culture and Tourism, Yangzhou 225127, China

**Keywords:** beef myofibrillar proteins, reduced-sodium meat products, konjac glucomannan, L-lysine, intermolecular interaction, gel properties

## Abstract

Reducing sodium in meat products is nutritionally desirable, but salt reduction often weakens myofibrillar protein gelation and reduces texture quality and water retention. This study investigated the effects of L-lysine (Lys) and konjac glucomannan (KGM) on the physicochemical properties, gel characteristics, and structural changes of beef myofibrillar protein (MP) gels under low-salt conditions. The results indicated that reducing NaCl from 0.6 to 0.2 M decreased water-holding capacity (WHC), increased cooking loss, and produced a loose gel network. Compared with the 0.2 M NaCl group, the combined Lys-KGM treatment increased WHC from 28.53% to 56.70% and reduced cooking loss to 22.19% (*p* < 0.05). Texture analysis showed that Lys-KGM increased hardness and springiness by 69.40% and 80.43%, respectively (*p* < 0.05). LF-NMR indicated a higher proportion of immobilized water and reduced water mobility in the combined treatment. Lys increased reactive sulfhydryl content and surface hydrophobicity, whereas KGM reduced surface hydrophobicity and enhanced water immobilization. Lys-KGM slightly but significantly decreased α-helix and increased β-sheet contents (*p* < 0.05), accompanied by changes in the relative contributions of intermolecular forces and a more continuous gel network. Molecular docking and molecular dynamics simulations provided supporting evidence for different interaction patterns between myosin and Lys/KGM. These results suggested that Lys and KGM might help maintain the quality of low-salt meat protein gels and provide a formulation basis for reduced-sodium meat products.

## 1. Introduction

The gelling properties of myofibrillar proteins (MP) largely determine the texture and water-holding capacity (WHC) of meat products [1,2,3]. In gel-type meat processing, 2–3% NaCl is commonly used to promote MP solubilization and heat-induced gel network formation, thereby helping maintain desirable sensory and textural properties [4,5]. Nevertheless, excessive sodium intake is closely related to hypertension and cardiovascular diseases [6]. The World Health Organization recommends reducing population salt intake by 30% and limiting daily salt intake to no more than 5 g for adults. As processed meat products contribute approximately 20–30% of total dietary sodium intake, the development of reduced-sodium meat products has received increasing attention [7]. However, NaCl reduction generally decreases MP solubilization and consequently weakens its gel-forming ability [8]. Maintaining MP gel quality under low-salt conditions remains a challenge.

L-Lysine (Lys) has been widely used in meat processing to enhance flavor and improve the functional properties of meat proteins [7]. Lys can enhance MP solubility and induce changes in the secondary and tertiary structures of MP [9,10]. These structural modifications may further regulate intermolecular interactions and thermal aggregation behavior of MP, thereby promoting gel formation under low-salt conditions [3,11,12,13]. Nevertheless, the improvement in gel quality by Lys alone remains limited under reduced-salt conditions, especially in maintaining gel integrity and water retention. Therefore, additional strategies are still required to improve low-salt MP gels.

Plant-derived polysaccharides have gained interest as functional ingredients for improving meat quality under low-salt conditions [14]. Konjac glucomannan (KGM), a water-soluble polysaccharide mainly composed of D-glucose and D-mannose linked by β-1,4-glycosidic bonds, possesses excellent hydration, thickening, and gel-forming properties [15,16]. KGM has been reported to increase the gel properties of MP systems by enhancing water retention, modulating protein aggregation behavior, and contributing to a denser and more stable gel structure during heating [17,18,19]. Therefore, the combination of Lys and KGM may provide complementary regulation for low-salt MP gelation through simultaneous modulation of protein molecular behavior and gel network structure. Such an ingredient-based strategy may help maintain texture and water retention in reduced-sodium meat products. However, the combined effects of Lys and KGM on intermolecular interactions, protein conformation, and gel network formation in low-salt MP systems remain unclear.

Therefore, this study aimed to evaluate the combined effects of Lys and KGM on the quality and structural characteristics of beef MP gels under low-salt conditions. WHC, cooking loss, water distribution, texture, rheological behavior, and microstructure were determined to assess gel quality, while sulfhydryl groups, surface hydrophobicity, FT-IR spectra, and intermolecular forces were used to characterize protein structural changes. Molecular docking and molecular dynamics simulations were further used as supporting tools to explore possible interactions between myosin and Lys/KGM. The results may support the combined use of Lys and KGM as functional ingredients to improve the quality of reduced-sodium meat products.

## 2. Results and Discussion

### 2.1. Cooking Loss and WHC

**Figure 1:**
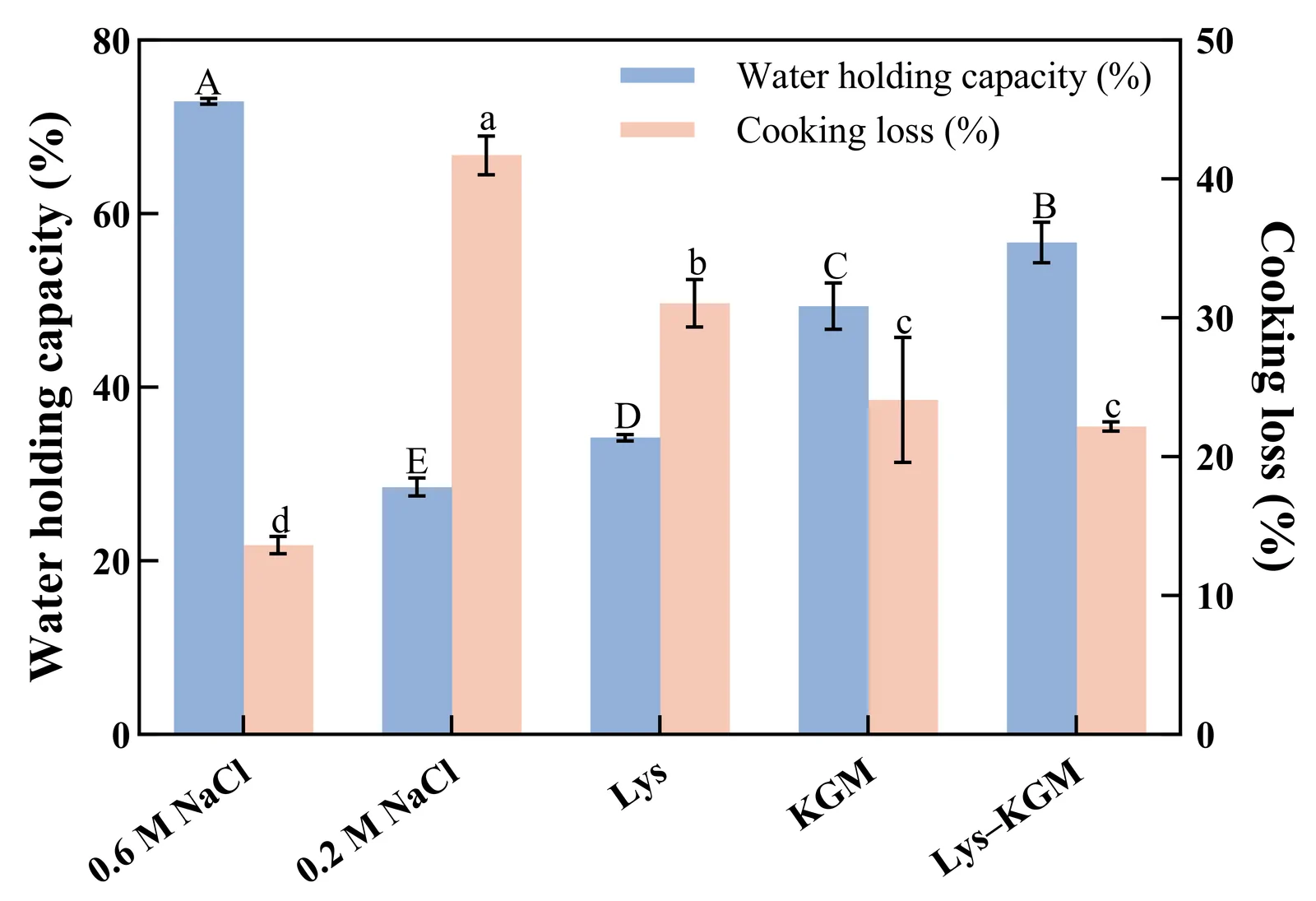
Water-holding capacity and cooking loss of MP gels under different treatments. The values are the mean ± standard deviation. Different capital letters indicate significant differences in water-holding capacity among different treatment groups. Different lowercase letters indicate that there are significant differences in cooking loss among different treatment groups (*p* < 0.05) (*n* = 3).

Cooking loss and WHC are commonly used to evaluate water retention and overall quality of heat-induced MP gels [20]. As presented in Figure 1, reducing NaCl from 0.6 M to 0.2 M increased cooking loss and decreased WHC, which might be attributed to insufficient MP solubilization and weakened intermolecular interactions under low-salt conditions, thereby impairing gel network formation during thermal gelation [8,21]. Compared with the 0.2 M NaCl group, Lys and/or KGM treatments significantly decreased cooking loss from 41.71% to 31.05%, 24.10%, and 22.19% and increased WHC from 28.53% to 34.21%, 49.38%, and 56.70%, respectively (*p* < 0.05). The Lys-KGM treatment showed the lowest cooking loss and the highest WHC, indicating enhanced water retention under low-salt conditions. Lys has been reported to improve MP solubility and promote protein unfolding and intermolecular interactions during thermal gelation, which contributed to greater water retention in the gel [22,23]. In contrast, KGM mainly improved hydration capacity and reduced water mobility during thermal gelation, thereby facilitating the development of a more uniform gel network [17,18].

### 2.2. Water Distribution

**Figure 2:**
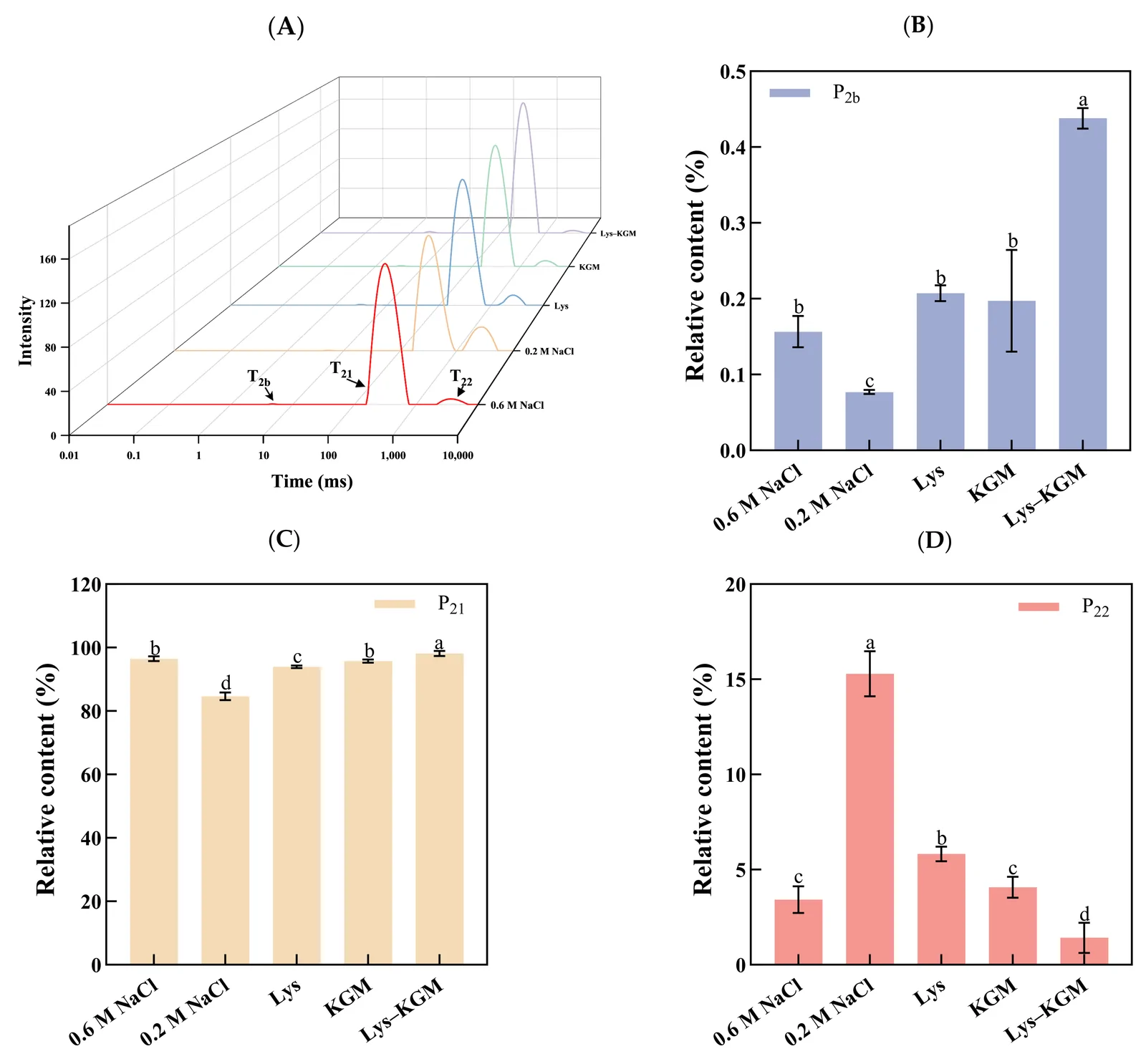
T_2_ relaxation time distribution (**A**) and water population proportions (**B**–**D**) of MP gels under different treatments. The values are the mean ± standard deviation. Different lowercase letters indicate significant differences among different treatment groups (*p* < 0.05) (*n* = 3).

LF-NMR relaxation behavior reflects water status in meat protein gels [24]. As presented in Figure 2A, three water populations were identified in all samples, including T_2b_ (1.96–6.83 ms), T_21_ (155.22–880.48 ms), and T_22_ (1245.88–7067.18 ms), which corresponded to bound water, immobilized water, and free water, respectively. Immobilized water was the predominant water population in the samples, suggesting that most water was retained in the gel network [25].

Peak area proportion (P_2_) reflected the relative distribution of water in the gel samples. As presented in Figure 2B–D, the 0.6 M NaCl group exhibited significantly higher relative contents of bound and immobilized water, accompanied by a lower relative content of free water, than the 0.2 M NaCl group (*p* < 0.05). This was consistent with Lin et al. [26], who found that higher NaCl levels shifted water toward the immobilized fraction and decreased the free water fraction in MP gels. In addition, the presence of Lys and/or KGM increased the relative contents of bound water and immobilized water while decreasing the relative content of mobile water. Notably, the Lys-KGM group exhibited the highest immobilized water content and the lowest mobile water content, reaching 98.15% and 1.42%, respectively (*p* < 0.05). This water distribution pattern agreed with the improved cooking loss and WHC results, suggesting better water retention in the gel network. The improved water distribution in the Lys-KGM group was likely associated with changes in MP structure and water immobilization. Lys could improve MP dispersion and promote protein unfolding under low-salt conditions, thereby facilitating intermolecular interaction during gel formation [10,22]. Unlike Lys, KGM mainly restricted water migration through its strong hydration capacity and stabilization of the protein-polysaccharide gel matrix [25,26]. Therefore, more water was retained as immobilized water in the Lys-KGM gel, which contributed to the improved WHC and reduced cooking loss.

### 2.3. Texture Properties Analysis of MP Gels

Textural properties of MP gels are strongly linked to gel network formation and are important indicators for evaluating the quality of gel-type meat products [1]. As presented in Table 1, the 0.6 M NaCl group showed higher hardness, springiness, cohesiveness, gumminess, and chewiness than the 0.2 M NaCl group (*p* < 0.05), indicating that salt reduction weakened the mechanical strength of MP gels. This might result from insufficient MP solubilization and limited intermolecular association at low salt levels, leading to weak gel network development [27]. Lys and/or KGM treatments improved the texture properties compared with the 0.2 M NaCl group. Hardness increased from 1.83 N to 2.20 N, 2.87 N, and 3.10 N in the Lys, KGM, and Lys-KGM groups, respectively (*p* < 0.05). A similar trend was observed for springiness, cohesiveness, gumminess, and chewiness. The Lys-treated group showed improved texture properties, which might be associated with the regulation of intermolecular interactions by Lys, thereby helping develop a more integrated protein network during heating [10,22]. KGM showed a stronger effect on hardness and chewiness, which was likely related to its contribution to structural stability and gel matrix continuity [18,25]. Notably, the Lys-KGM group showed the highest hardness, even exceeding that of the 0.6 M NaCl group (*p* < 0.05), indicating a marked improvement in gel strength. The results were consistent with the higher immobilized water content, lower cooking loss, and higher WHC observed in the Lys-KGM group.

### 2.4. Rheological Properties

**Figure 3:**
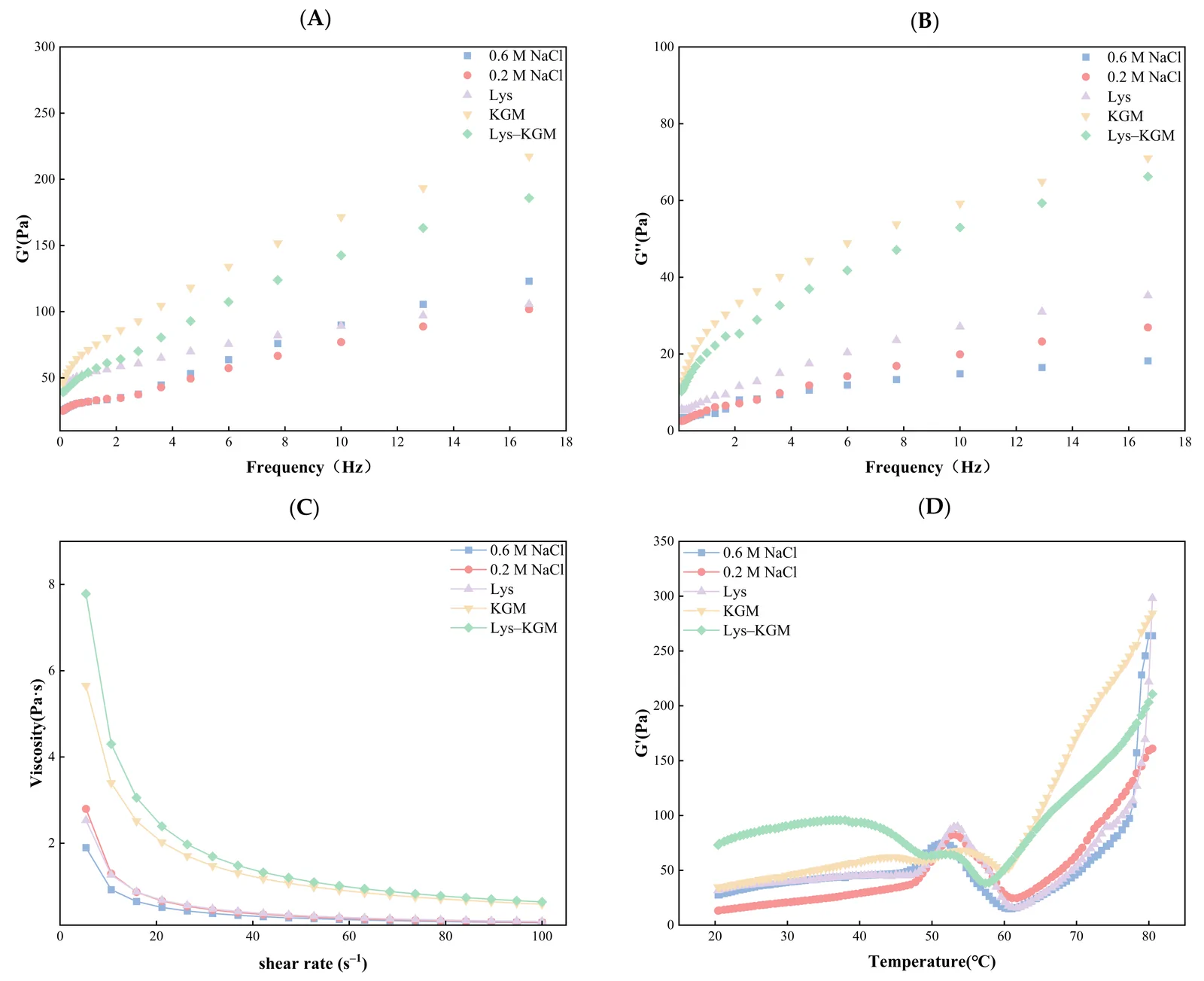

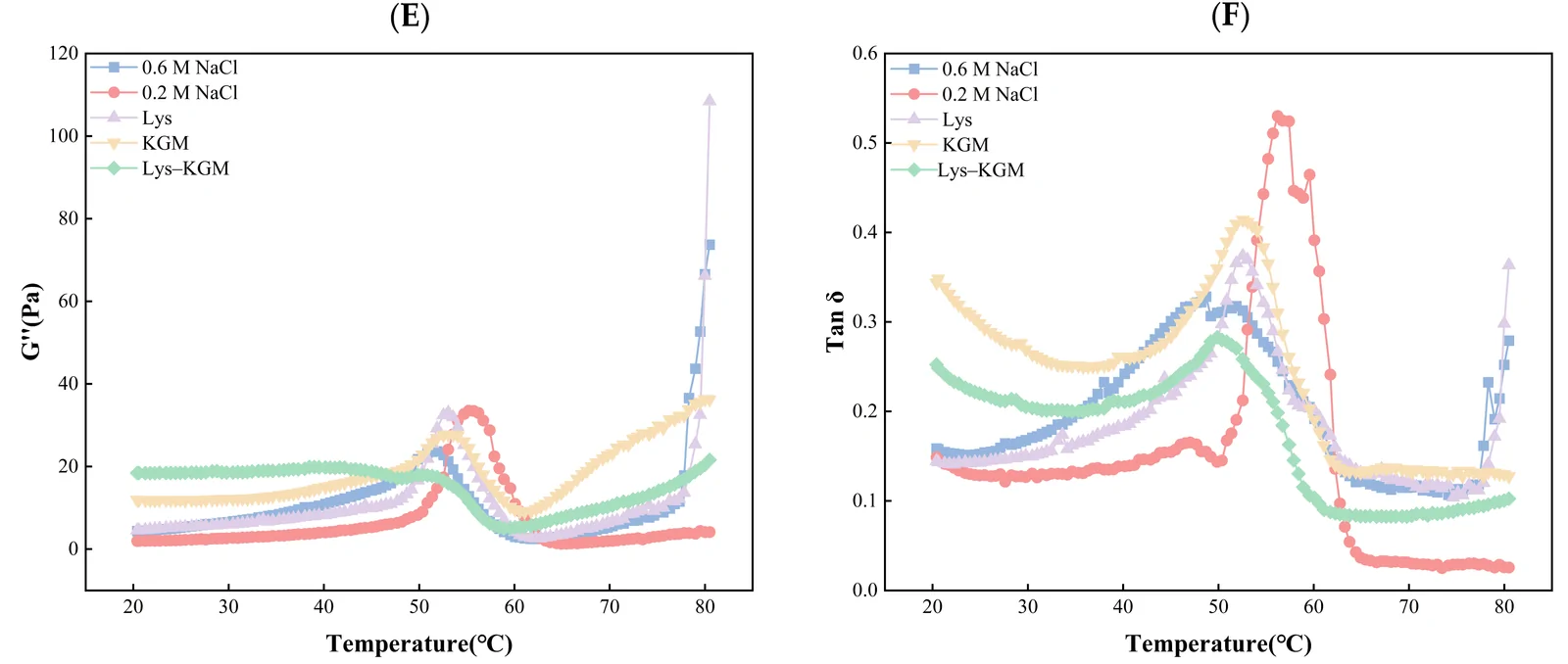
Frequency sweep (**A**,**B**), apparent viscosity (**C**), and temperature sweep (**D**–**F**) of MP systems under different treatments.

Dynamic rheology was used to evaluate the viscoelastic behavior and thermal gelation characteristics of MP suspensions. As presented in Figure 3A,B, both G′ and G″ values increased with increasing frequency in all groups, indicating frequency-dependent viscoelastic behavior. Across the tested frequency range, G′ values were consistently higher than G″ values, suggesting that all samples exhibited elastic-dominant viscoelastic behavior. The storage modulus reflected elastic energy storage during deformation, whereas the loss modulus reflected viscous energy dissipation [28]. The 0.6 M NaCl group showed higher G′ and G″ values than the 0.2 M NaCl group, which was mainly attributed to improved MP solubilization and enhanced protein association under high-salt conditions. Lys and/or KGM increased the viscoelastic response of low-salt MP suspensions. The Lys group showed higher G′ and G″ values, suggesting that Lys helped improve the elastic network of MP gels under low-salt conditions. This might be related to the ability of Lys to improve myosin dispersion and promote protein association during gelation [22,23]. KGM-containing groups showed more significant increases in G′ and G″, especially in the KGM group. This result indicated that KGM strongly contributed to the elastic and viscous behavior of samples. The improvement was likely associated with the hydrated polysaccharide chains of KGM, which strengthened matrix continuity and increased the viscoelastic response of the gel network [29,30]. Nevertheless, the Lys-KGM group showed lower G′ and G″ values than the KGM group, indicating that the combined treatment did not further enhance viscoelasticity through the thickening effect of KGM alone.

All samples showed shear-thinning behavior, and apparent viscosity decreased with increasing shear rate (Figure 3C and Table 2). The Power-Law model was further applied to quantitatively characterize the flow behavior of MP suspensions. The flow behavior index (*n*) values of all groups were below 1 (0.060–0.221), confirming their typical pseudoplastic characteristics. The low *n* values indicated that the internal structures of the suspensions were highly sensitive to shear deformation, resulting in a pronounced reduction in viscosity under increased shear rates. Among all samples, the 0.2 M NaCl group exhibited the lowest *n* value (0.060), suggesting the strongest shear dependence and easier disruption of internal interactions during shearing. In addition, the Power-Law model showed good fitting performance for all samples, with R^2^ values ranging from 0.981 to 0.999. The KGM group and the Lys-KGM group exhibited the highest fitting accuracy (R^2^ = 0.999), indicating that the Power-Law model effectively described their shear-dependent flow behavior. This trend might be due to the gradual disruption of weak physical interactions in the gel sols under shear [31]. Apparent viscosity mainly reflected the flow resistance of MP dispersions before gel formation. Lys caused a slight increase in viscosity, indicating a limited thickening effect in the low-salt MP system. KGM and Lys-KGM markedly increased apparent viscosity, especially at low shear rates. This increase was mainly related to the strong hydration capacity and intermolecular entanglement of KGM chains, which increased flow resistance in the continuous phase [29,32]. The Lys-KGM group maintained high viscosity, suggesting that KGM mainly determined the flow behavior of samples, whereas Lys contributed more to MP dispersion than to viscosity increase.

The temperature sweep results are shown in Figure 3D,E. During heating, G′ showed a typical three-stage pattern, including an initial increase, a temporary decrease, and a sharp increase at higher temperatures. This pattern was associated with protein unfolding, structural rearrangement, and heat-induced aggregation during MP gel formation [33,34,35]. Lys, KGM, and Lys-KGM treatments increased the final G′ value, indicating improved heat-induced gelation under low-salt conditions. The KGM group exhibited a relatively stable G′ profile during heating, with G′ values remaining generally higher than those of the 0.2 M NaCl group. This result suggested that KGM strongly contributed to elastic structure development during heating, probably due to its hydration effects and matrix-thickening effects. However, the Lys-KGM group showed better WHC and texture properties than the individual treatments, although its final G′ was not the highest. This indicated that the quality improvement in the Lys-KGM gel was not determined only by elastic modulus, but also by water immobilization and network organization.

As shown in Figure 3F, tan δ values remained below 1 in all groups, confirming the solid-like behavior of MP gels. The decrease in tan δ at the later heating stage suggested that elastic properties gradually became dominant during gel formation. KGM mainly increased viscosity and improved the viscoelastic properties of samples, whereas Lys showed a weaker effect on rheological behavior. These changes were in agreement with the improved water retention and texture observed in the Lys-KGM gels.

### 2.5. Microstructure of MP Gels

**Figure 4:**
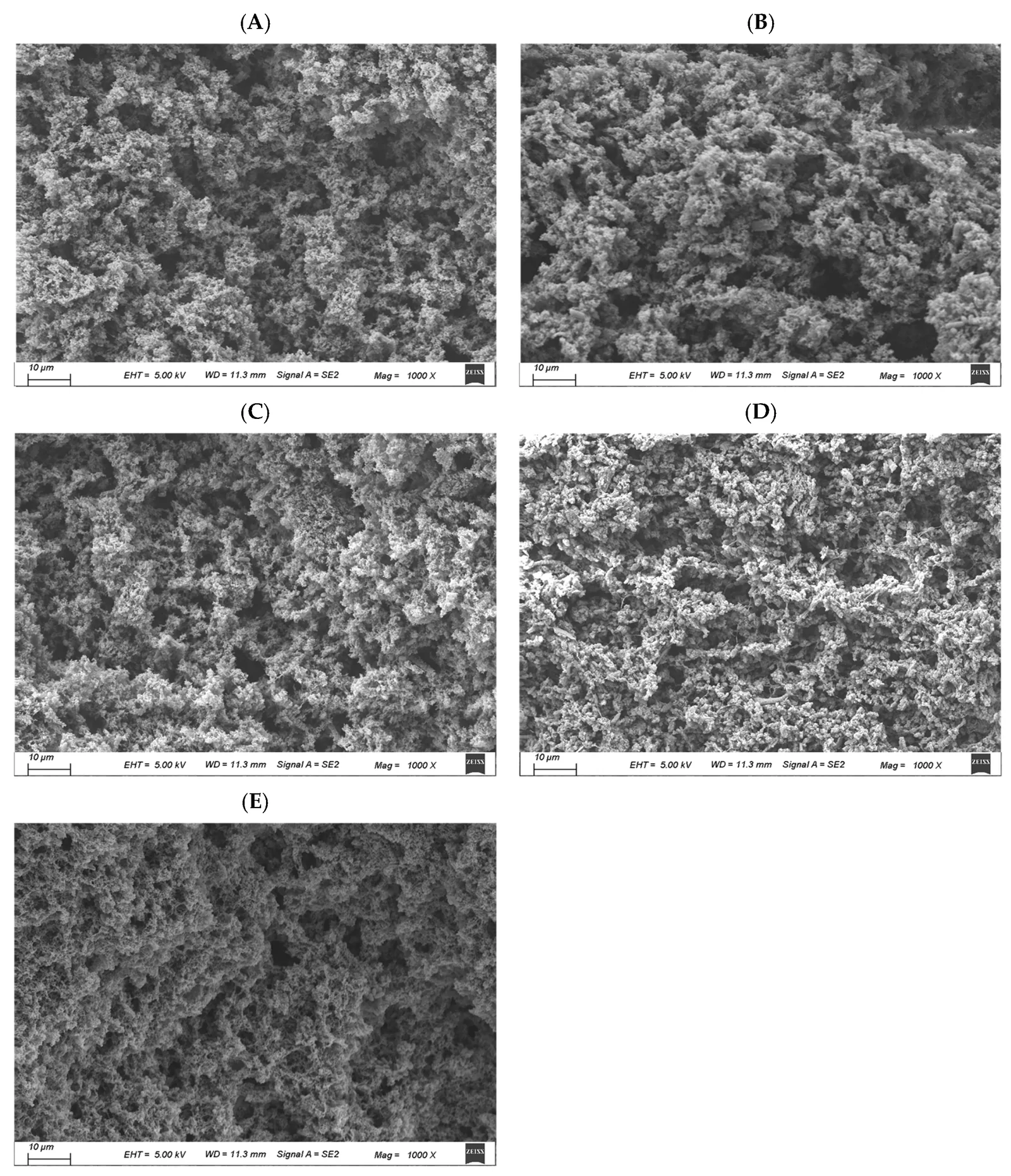
SEM micrographs of MP gels under different treatments: 0.6 M NaCl (**A**), 0.2 M NaCl (**B**), Lys (**C**), KGM (**D**), and Lys-KGM (**E**).

The microstructure of MP gels is shown in Figure 4. The 0.6 M NaCl group formed a dense and continuous network with small pores, whereas the 0.2 M NaCl group showed an irregular structure with large voids and weak continuity. This weak structure might result from poor MP solubilization and limited protein association at low salt levels, leading to insufficient network formation during heating [21,27,34]. The microstructural changes were consistent with the high cooking loss, low WHC, reduced immobilized water content, and weak texture observed in the 0.2 M NaCl group. After Lys addition, the gel network became more continuous with smaller pores, although some irregular pores were still present. This suggested that Lys partially improved the microstructure of low-salt MP gels. Previous studies reported that Lys could improve myosin dispersion and regulate protein association under reduced-salt conditions, thereby facilitating the development of a more integrated protein network [22,23]. The KGM group showed a more homogeneous and continuous structure than the Lys group, with fewer large pores and improved matrix integrity. This result suggested that KGM contributed more strongly to network organization. The high hydration capacity and chain interaction of KGM might help stabilize the protein-polysaccharide matrix and reduce structural defects during gel formation [17,18,29,30]. Notably, the Lys-KGM group exhibited the most uniform and continuous network among all low-salt groups, with fewer large cavities and better structural integrity. This morphology suggested that the Lys-KGM treatment improved the organization of the low-salt MP gel network. The improved microstructure was consistent with the significantly higher WHC, higher immobilized water content, and improved hardness observed in the Lys-KGM group.

### 2.6. T-SH and R-SH Content

Sulfhydryl groups are sensitive indicators of MP conformational changes and thiol-related reactions during protein structural transition [8]. No significant difference was observed in T-SH content among the treatment groups (*p* > 0.05; Table 3), indicating that NaCl reduction and Lys/KGM addition did not markedly change the total amount of sulfhydryl groups in MP. In contrast, R-SH content changed significantly among treatments *(p* < 0.05). Compared with the 0.6 M NaCl group, R-SH content decreased from 1.81 to 1.59 μmol/g in the 0.2 M NaCl group (*p* < 0.05). This decrease might be related to insufficient MP solubilization at low salt levels, which reduced the exposure of buried sulfhydryl groups [27,36]. After Lys addition, R-SH content increased from 1.59 to 1.80 μmol/g, which was close to the value of the 0.6 M NaCl group (*p* < 0.05). Lys could promote MP dispersion and protein unfolding, leading to increased exposure of sulfhydryl groups [22,23]. R-SH content also increased to 1.73 μmol/g in the presence of KGM (*p* < 0.05), which might be related to MP-KGM interactions that altered protein conformation and improved sulfhydryl exposure [18,37].

### 2.7. Analysis of the Surface Hydrophobicity of MP

The effect of Lys and/or KGM on the surface hydrophobicity of MP is shown in Table 3. The BPB binding value of 0.2 M NaCl group decreased from 21.90 to 18.30 μg BPB/mg protein compared to the 0.6 M NaCl group (*p* < 0.05), resulting from insufficient MP solubilization and limited conformational unfolding of MP at low-salt concentrations [22,38]. Lys increased BPB binding to 19.17 μg BPB/mg protein compared with the 0.2 M NaCl group (*p* < 0.05), which might be attributed to the increased MP solubility and protein unfolding induced by Lys under low-salt conditions [10,22]. In contrast, KGM treatment markedly reduced BPB binding to 10.97 μg BPB/mg protein (*p* < 0.05). This decrease might result from MP-KGM interactions that altered the protein surface environment and limited BPB binding to hydrophobic sites [18]. The Lys-KGM group showed higher BPB binding than the KGM group but lower values than the 0.2 M NaCl and Lys groups (*p* < 0.05), suggesting that Lys partly reduced the effect of KGM on hydrophobic exposure.

### 2.8. FT-IR of MP Gels

**Figure 5:**
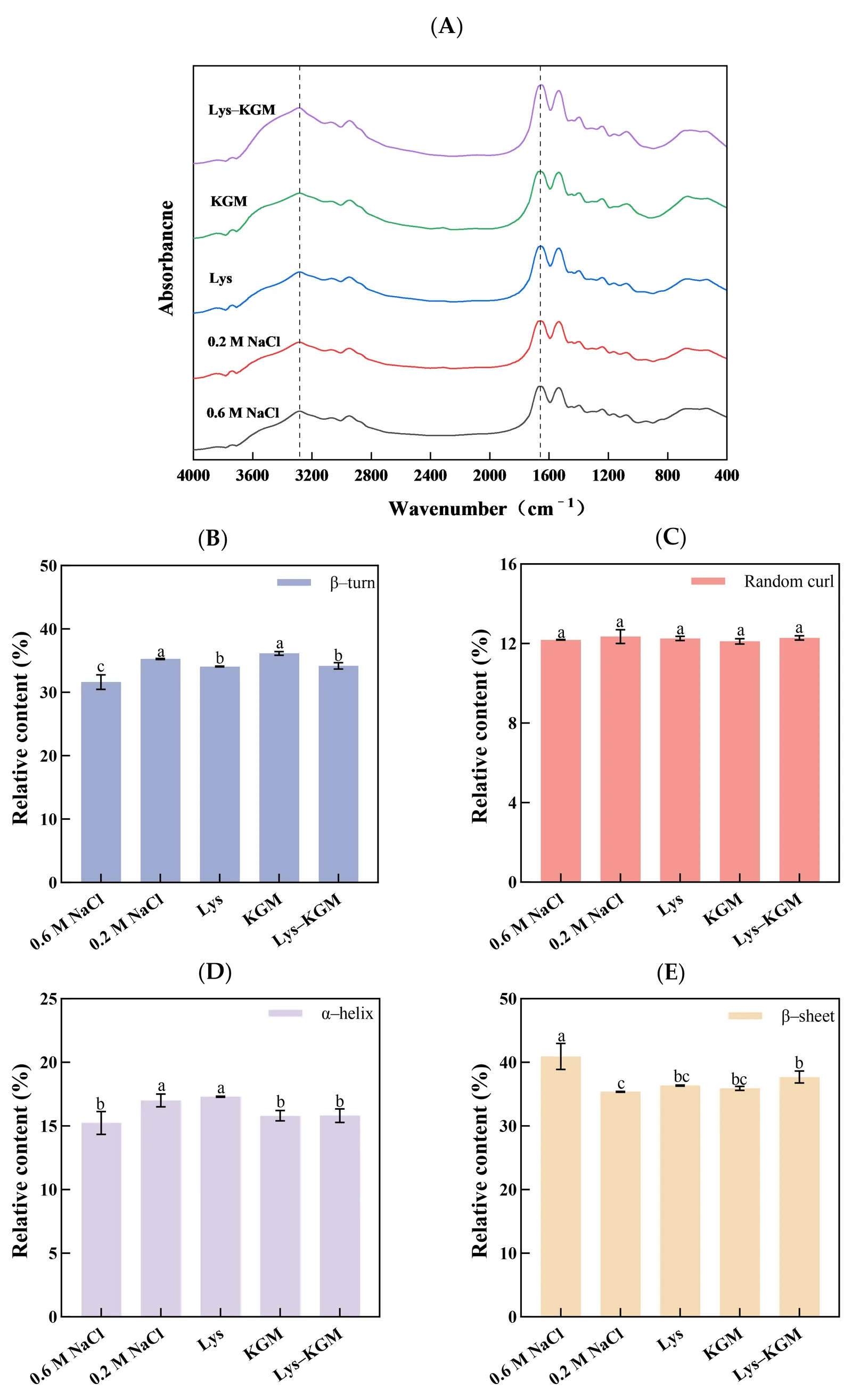

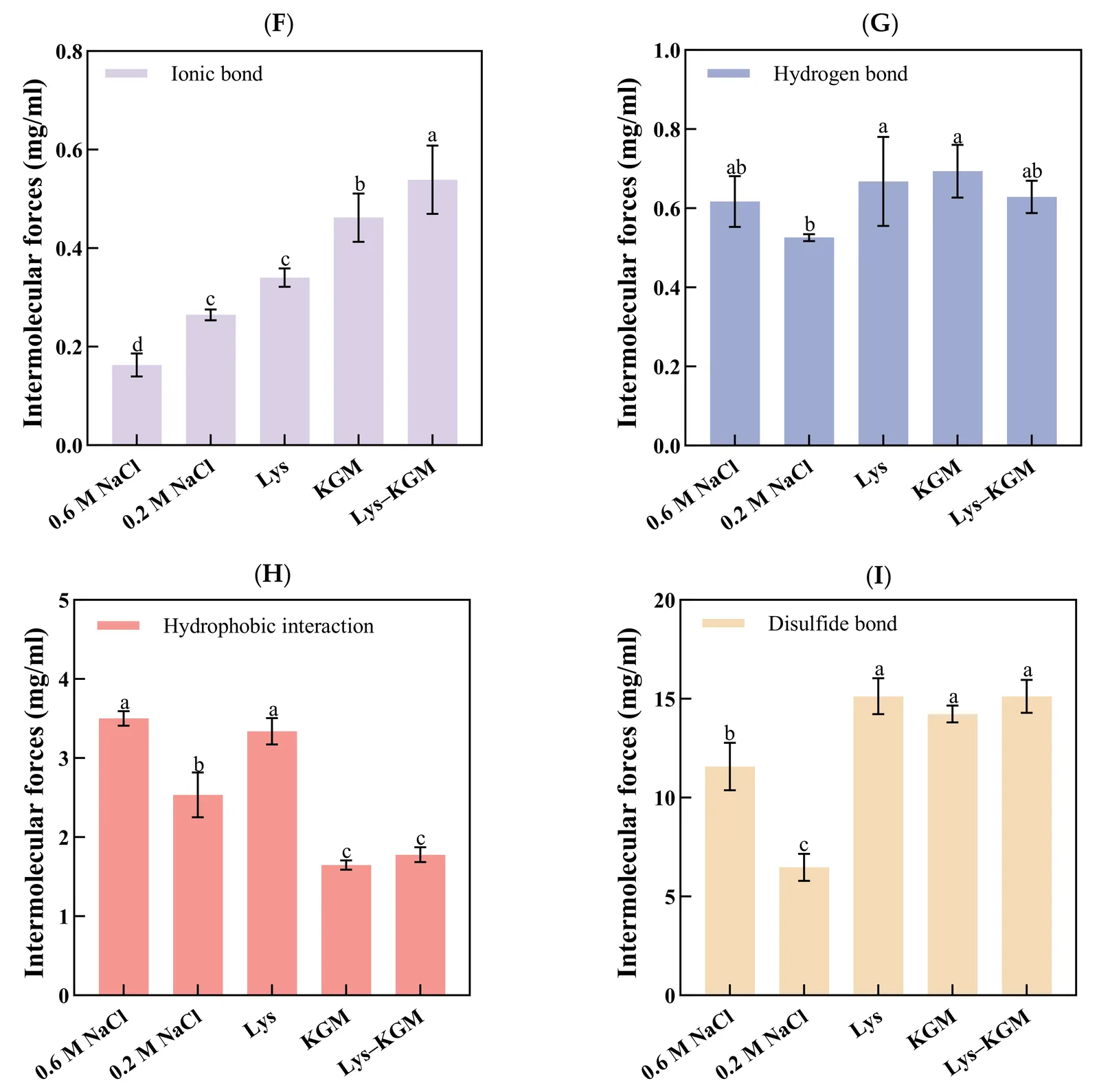
FT-IR spectra (**A**), secondary structure composition (**B**–**E**), and intermolecular forces (**F**–**I**) of MP gels under different treatments. The values are the mean ± standard deviation. Different lowercase letters indicate significant differences among different treatment groups (*p* < 0.05) (*n* = 3).

FT-IR analysis was performed to characterize changes in the secondary structure and hydrogen-bonding environment of MP gels. As shown in Figure 5A, no new absorption peaks were detected after Lys, KGM, or Lys-KGM treatment, suggesting that no detectable new covalent functional groups were formed in the MP gels. The amide A band, mainly associated with N–H stretching vibrations, showed a slight shift from 3281 to 3282 cm^−1^ in the Lys-KGM group. This shift suggested a possible change in the hydrogen-bonding environment of MP [18]. The amide I band at 1600–1700 cm^−1^ was further analyzed because it is sensitive to changes in protein secondary structure. As presented in Figure 5B–E, the 0.2 M NaCl group showed higher α-helix and β-turn contents, but lower β-sheet content than the 0.6 M NaCl group (*p* < 0.05). This suggested that salt reduction changed the secondary structure distribution of MP gels and limited the formation of β-sheet structures, which might be related to insufficient MP unfolding and weakened intermolecular interaction at low salt concentration. Compared with the 0.2 M NaCl group, Lys treatment did not significantly alter α-helix, β-sheet, or random coil contents (*p* > 0.05), although β-turn content decreased significantly (*p* < 0.05). The presence of KGM decreased α-helix content (*p* < 0.05). Compared with the 0.2 M NaCl group, the Lys-KGM group showed a small but significant decrease in α-helix content and a corresponding increase in β-sheet content (*p* < 0.05). These differences might be associated with hydrogen bonding and other protein-polysaccharide interactions involving KGM and MP [18,37]. The increase in β-sheet content might be related to intermolecular association during gelation [39]. These FT-IR findings were consistent with the improved WHC, water immobilization, texture, and microstructure observed in the Lys-KGM group.

### 2.9. Molecular Forces in MP Gels

Intermolecular forces play an important role for the formation and stability of MP gel networks [4]. Ionic bonds, hydrogen bonds, hydrophobic interactions, and disulfide bonds were involved in the formation of MP gels (Figure 5F–I), among which hydrophobic interactions and disulfide bonds were predominant. The 0.2 M NaCl group showed significantly lower hydrogen bonds, hydrophobic interactions, and disulfide bonds (*p* < 0.05) than the 0.6 M NaCl group, indicating that salt reduction weakened intermolecular association during gel formation. This result aligned with the lower WHC, weaker textural properties, and looser microstructure observed in the low-salt group [27,34]. In contrast, ionic bonds increased significantly after salt reduction (*p* < 0.05), probably due to reduced ionic shielding. Lys and/or KGM treatments significantly changed intermolecular force distribution compared with the 0.2 M NaCl group. Lys treatment increased the estimated contributions attributed to hydrogen bonds, hydrophobic interactions, and disulfide bonds (*p* < 0.05), suggesting enhanced intermolecular interactions during gel formation. This agreed with the increased R-SH content and surface hydrophobicity. KGM treatment increased ionic bonds and hydrogen bonds (*p* < 0.05), which might result from MP-KGM interactions and the hydrogen bonding capacity of hydroxyl groups in KGM [18,37]. This agreed with Cao et al. [4], who reported that polysaccharides changed intermolecular interactions and improved gel stability. Meanwhile, KGM markedly reduced hydrophobic interactions (*p* < 0.05), which agreed with the lower surface hydrophobicity observed previously. The Lys-KGM group showed the highest estimated contribution attributed to ionic bonds and maintained a high estimated contribution attributed to disulfide bonds among the low-salt groups (*p* < 0.05), indicating that the combined treatment altered the intermolecular force distribution during gel formation. These changes were consistent with the higher WHC, increased immobilized water content, improved texture, and more continuous microstructure observed in the Lys-KGM group.

### 2.10. Molecular Docking of KGM/Lys with MP

Molecular docking simulations were used to predict the preferred binding conformation and affinity between ligands and macromolecular receptors. When combined with molecular dynamics simulations, they can further provide information on the stability and dynamic behavior of the interaction system [40,41]. As the main component of MP, myosin was selected as the receptor to examine its interactions with Lys and KGM [42]. The binding energies of KGM-myosin and Lys-myosin were −8.4 and −7.7 kcal/mol, respectively. The negative binding energies suggested that both KGM and Lys could spontaneously interact with myosin. The more negative value of KGM-myosin indicated a stronger predicted binding affinity than Lys-myosin [42,43].

**Figure 6:**
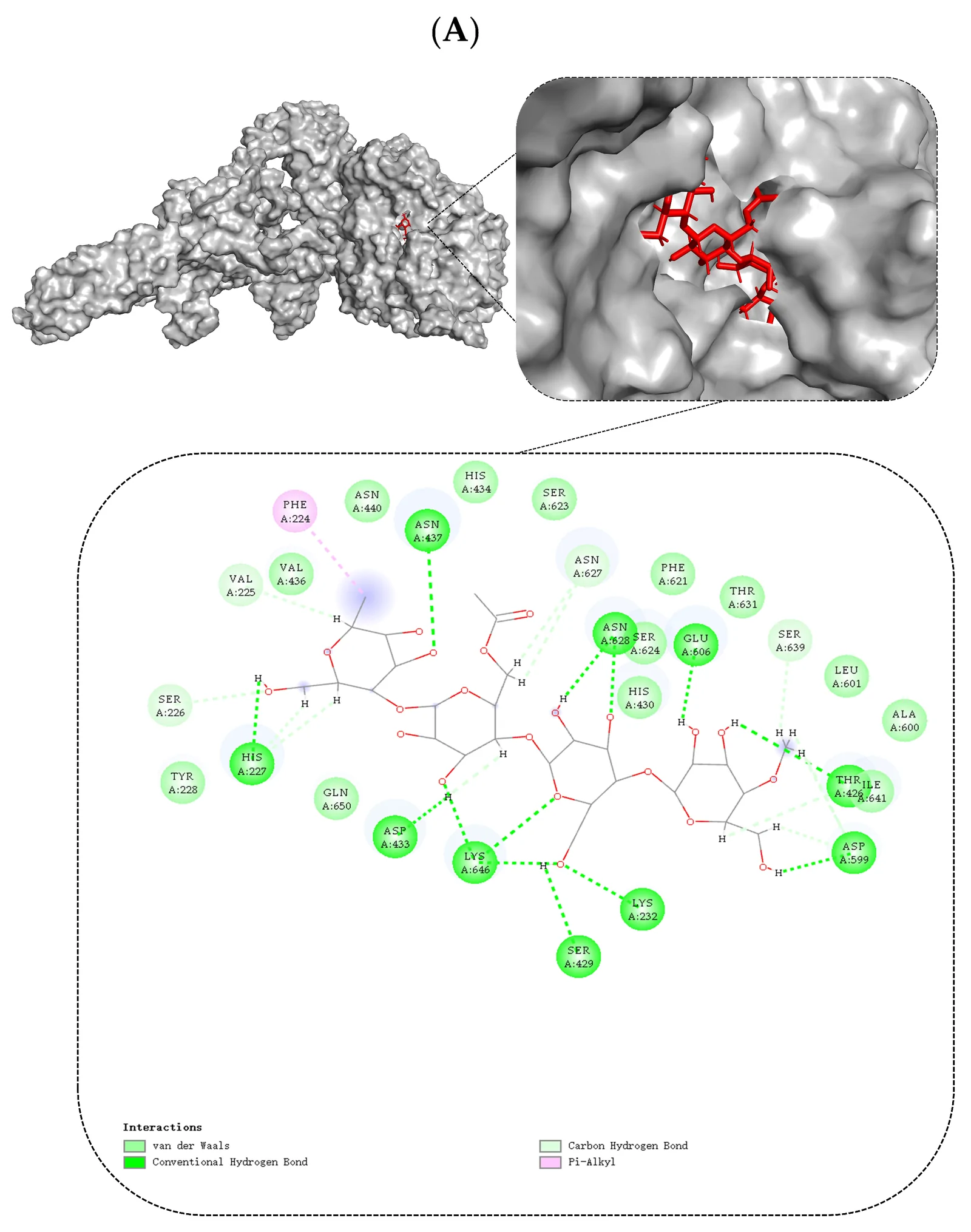

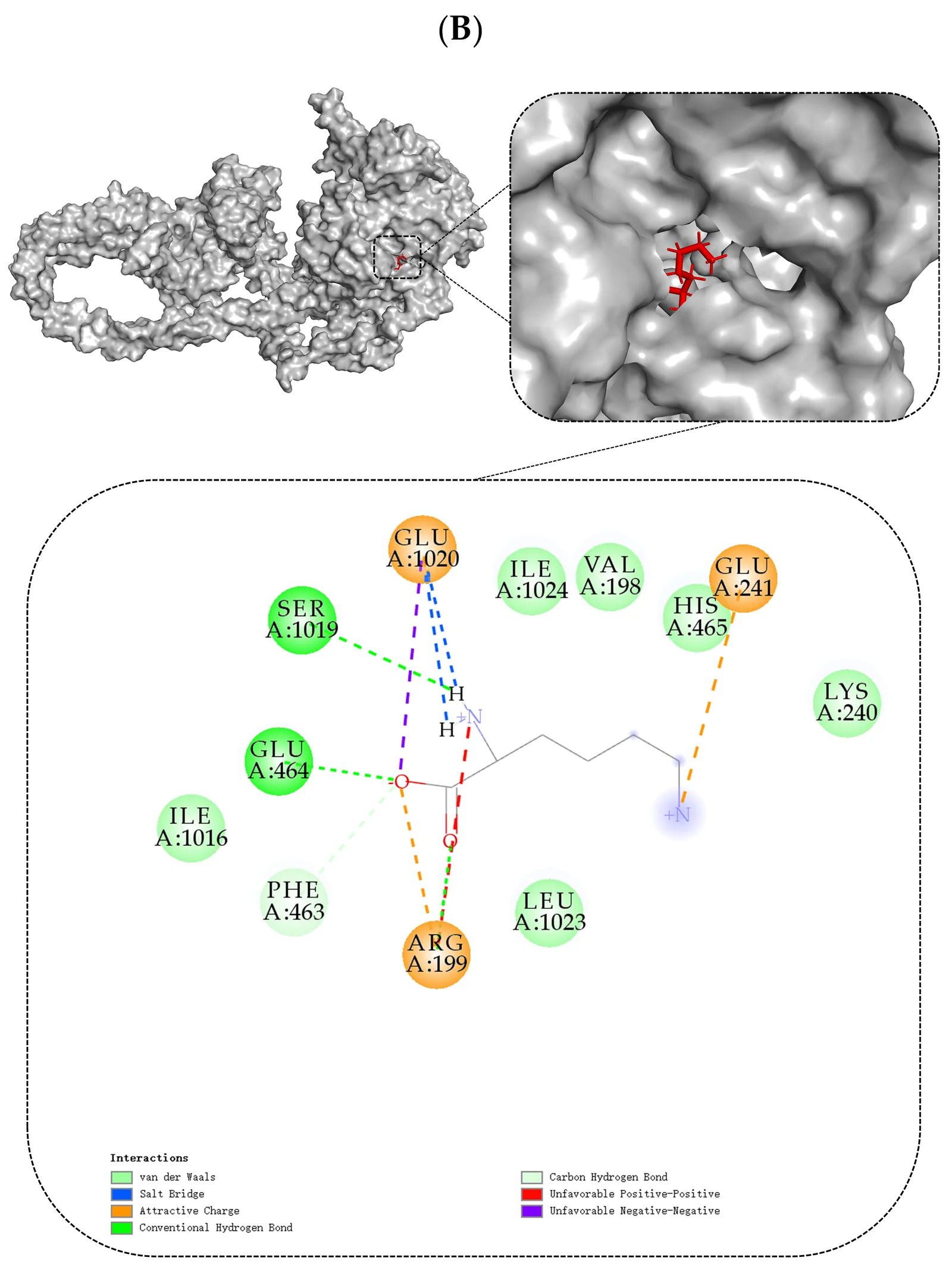

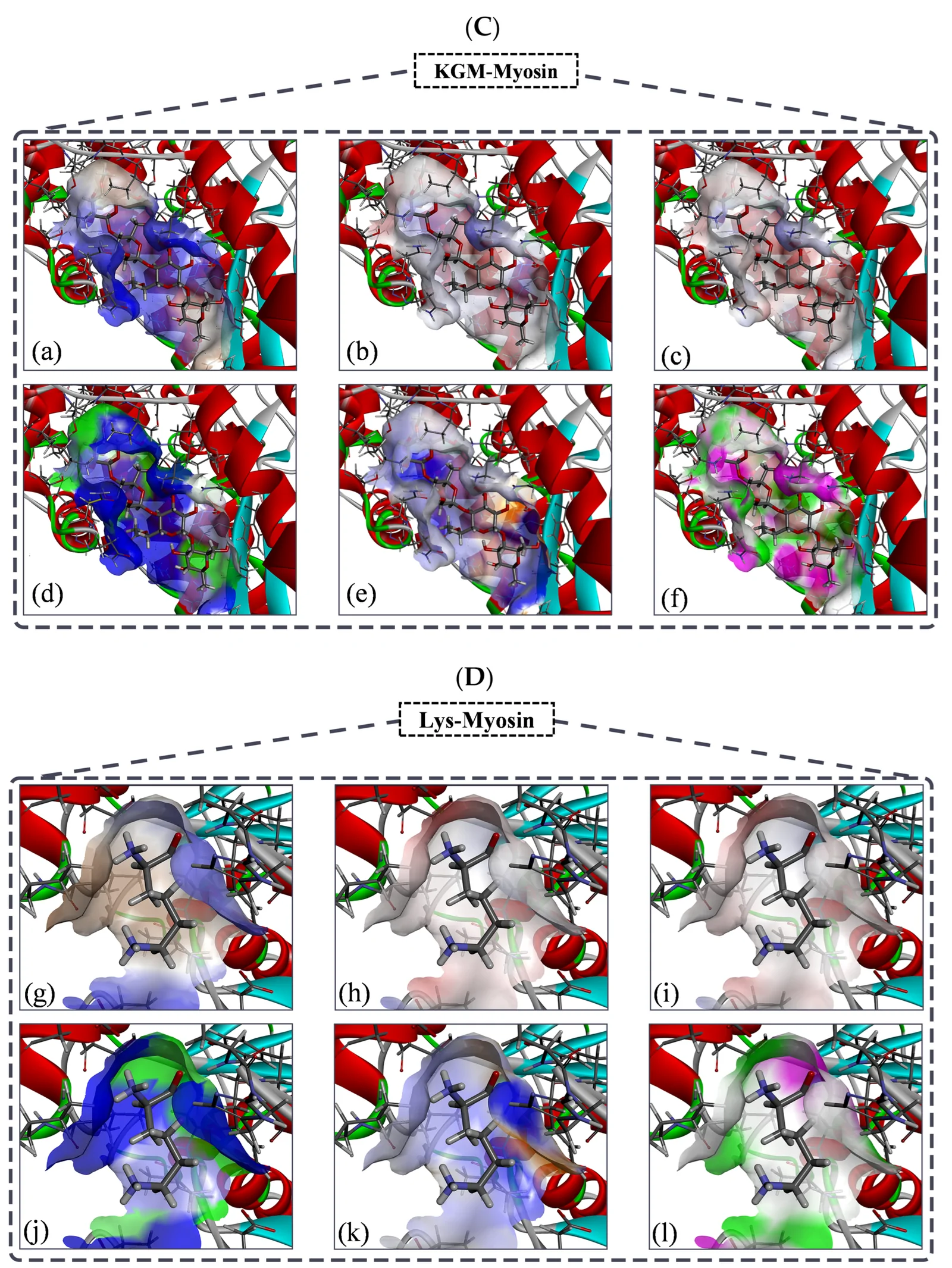
Molecular docking analysis of the interaction between Myosin and KGM/Lys. The 2D structures of myosin docked with KGM and Lys are shown in panels (**A**,**B**), and the 3D structures are (**C**,**D**). In panel (**C**), subgraphs (**a**–**f**) represent hydrophobic contour, ionizability contour, interpolated charge contour, secondary hydrophobic view, secondary charge view, and hydrogen-bond field of the KGM-myosin complex. In panel (**D**), subgraphs (**g**–**l**) display the above-mentioned six physic-chemical fields for the L-lysine-myosin complex. Different surface colors denote distinct molecular properties. For hydrophobic contour, blue regions favor hydrophobic interactions. For electrostatic-related fields, blue corresponds to positive potential and red corresponds to negative potential. For hydrogen-bond field, green is favorable for hydrogen-bond donors, and magenta/purple favors hydrogen-bond acceptors.

As presented in Figure 6A,B, KGM and Lys bound to different sites of myosin. KGM showed more binding residues than Lys, including PHE224, VAL225, SER226, HIS227, TYR228, ASN440, HIS434, ASN437, PHE621, SER623, SER624, ASN627, and ASN628. Additional residues, including HIS430, GLU606, THR631, SER639, LEU601, ALA600, THR426, ILE641, ASP599, LYS232, SER429, LYS646, ASP433, and GLN650, were also involved in KGM-myosin interactions. In contrast, Lys mainly interacted with residues including ILE1016, GLU464, SER1019, GLU1020, ILE1024, VAL198, HIS465, GLU241, LYS240, LEU1023, ARG199, and PHE463. As shown in Figure 6C,D, the surface interaction maps further confirmed that KGM and Lys occupied different local regions of myosin and exhibited different interaction patterns. KGM interacted with myosin mainly through conventional hydrogen bonds, carbon hydrogen bonds, and van der Waals forces, with limited π-alkyl/hydrophobic contacts. These interactions were probably related to the abundant hydroxyl groups and flexible chain structure of KGM, which favored contacts with polar residues of myosin. Lys interacted with myosin mainly through salt bridges, charge interactions, hydrogen bonds, carbon hydrogen bonds, and van der Waals forces. The presence of unfavorable charge interactions indicated that electrostatic effects in the Lys-myosin complex were not fully favorable. Thus, KGM and Lys appeared to interact with myosin through different binding patterns. This agreed with previous studies showing that KGM could interact with proteins through hydrogen bonds and other non-covalent forces, thereby affecting protein conformation and gel properties [16,37,44]. Lys has also been reported to improve functional properties of MP by changing protein conformation and molecular interactions [12]. Taken together, the docking results showed that both KGM and Lys could bind to myosin, but with different binding sites and interaction types. KGM had stronger predicted affinity and involved more interaction residues, whereas Lys was more affected by charge-related interactions. These findings supported the different roles of KGM and Lys in regulating MP conformation and intermolecular interactions, which was in line with the FT-IR and molecular force results.

### 2.11. Molecular Dynamics Simulations

#### 2.11.1. RMSD

**Figure 7:**
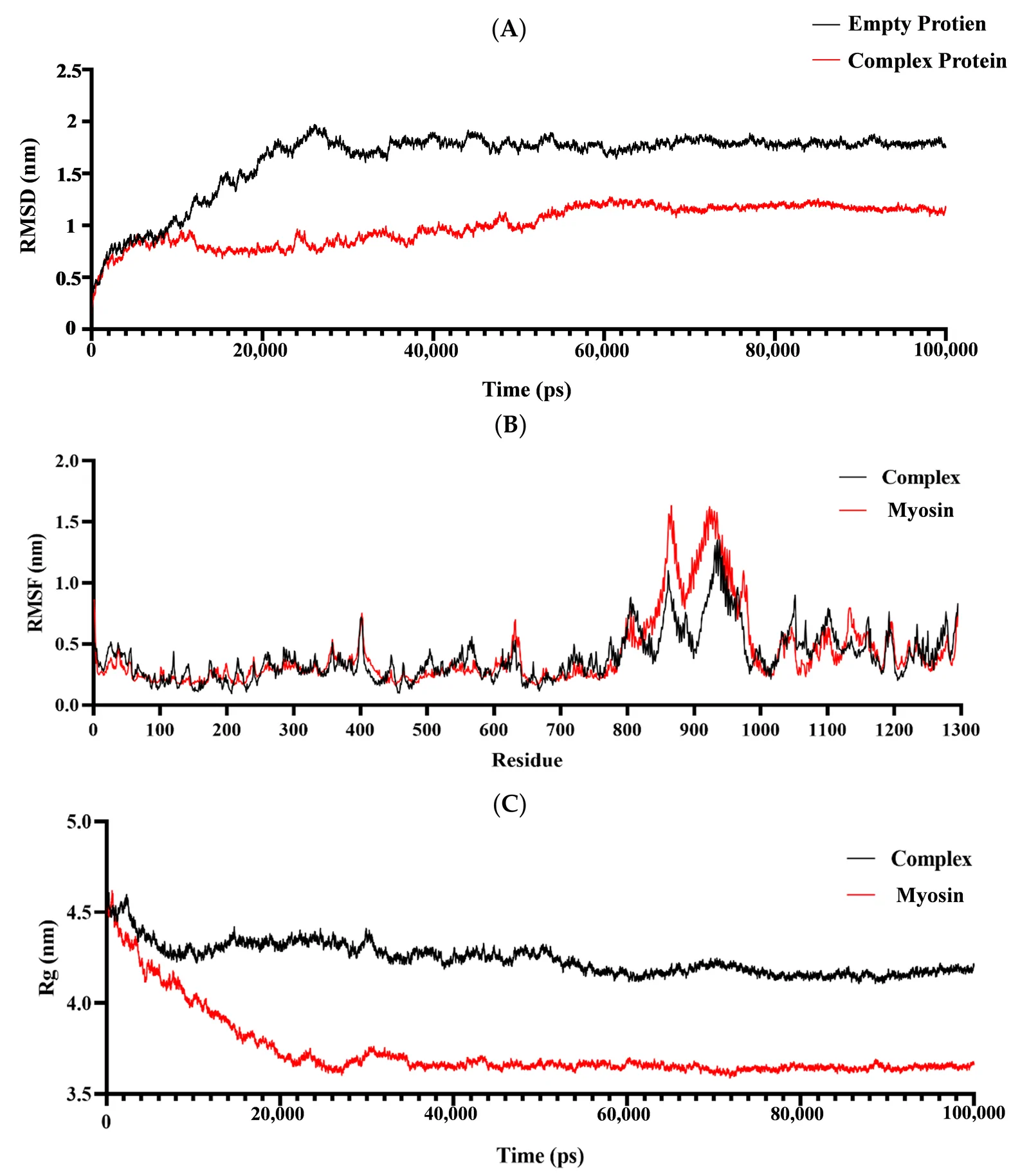

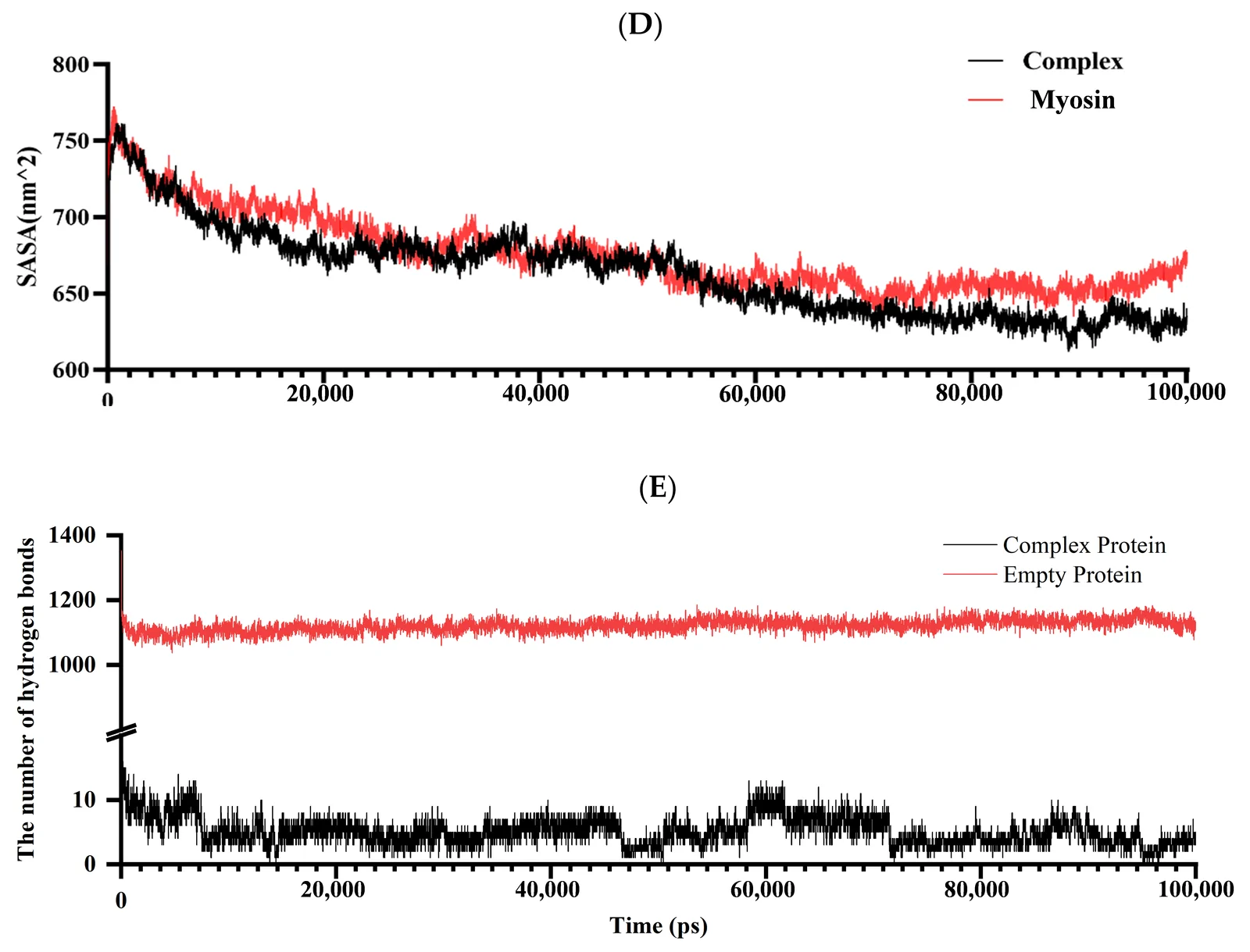
The molecular dynamics simulation of myosin and Lys-KGM-Myosin complex (**A**–**E**). The RMSD (**A**), RMSF (**B**), Rg (**C**), and solvent accessible surface area (SASA, (**D**)) of the myosin and Lys-KGM-Myosin complex. Number of hydrogen bonds in Myosin and Lys-KGM-Myosin complex (**E**).

RMSD was used to follow the backbone deviation of myosin during simulation and to assess the stability of the protein-ligand complex [45]. As shown in Figure 7A, RMSD values increased quickly at the beginning and then became stable, indicating that the systems gradually reached equilibrium. After 10 ns, the Lys-KGM-myosin complex showed lower RMSD values than free myosin, suggesting that Lys-KGM binding helped stabilize myosin during simulation [4,40]. This lower RMSD indicated reduced structural fluctuation after ligand binding.

#### 2.11.2. RMSF

RMSF was used to analyze the flexibility of amino acid residues during simulation [46]. RMSF values varied among different residue regions (Figure 7B), indicating differences in local flexibility along the myosin chain. Compared with free myosin, the Lys-KGM-myosin complex showed lower RMSF values in several regions, suggesting reduced residue mobility after ligand binding [47,48]. However, some residues still showed relatively high RMSF values, indicating that local flexibility was retained during simulation. These results suggested that Lys-KGM mainly affected local residue movement rather than restricting the whole protein structure.

#### 2.11.3. Rg, SASA, and Hydrogen Bonds

Rg reflects the compactness of protein structure during simulation [46]. As illustrated in Figure 7C, the Rg value decreased rapidly at the initial stage and then tended to stabilize, indicating that the Lys-KGM-myosin complex became more compact after structural adjustment. This result suggested that Lys-KGM binding helped maintain a compact conformation of myosin rather than inducing structural expansion.

SASA reflects the solvent-accessible surface area of proteins [49]. As shown in Figure 7D, the SASA value gradually decreased and then remained relatively stable, indicating that the solvent-exposed surface area of myosin was reduced after complex formation. This change suggested that part of the myosin surface became less accessible to solvent, which was consistent with the reduced surface exposure observed in KGM-containing systems [18].

Hydrogen bonds are important non-covalent interactions involved in protein-ligand binding [41,50]. As illustrated in Figure 7E, the number of hydrogen bonds fluctuated within a relatively stable range during simulation, suggesting that hydrogen bonding maintained the interaction between Lys-KGM and myosin. However, the hydrogen bond profile should be interpreted as evidence of dynamic binding stability rather than direct proof of gel network formation.

#### 2.11.4. Binding Energy and Free Energy Landscape

**Figure 8:**
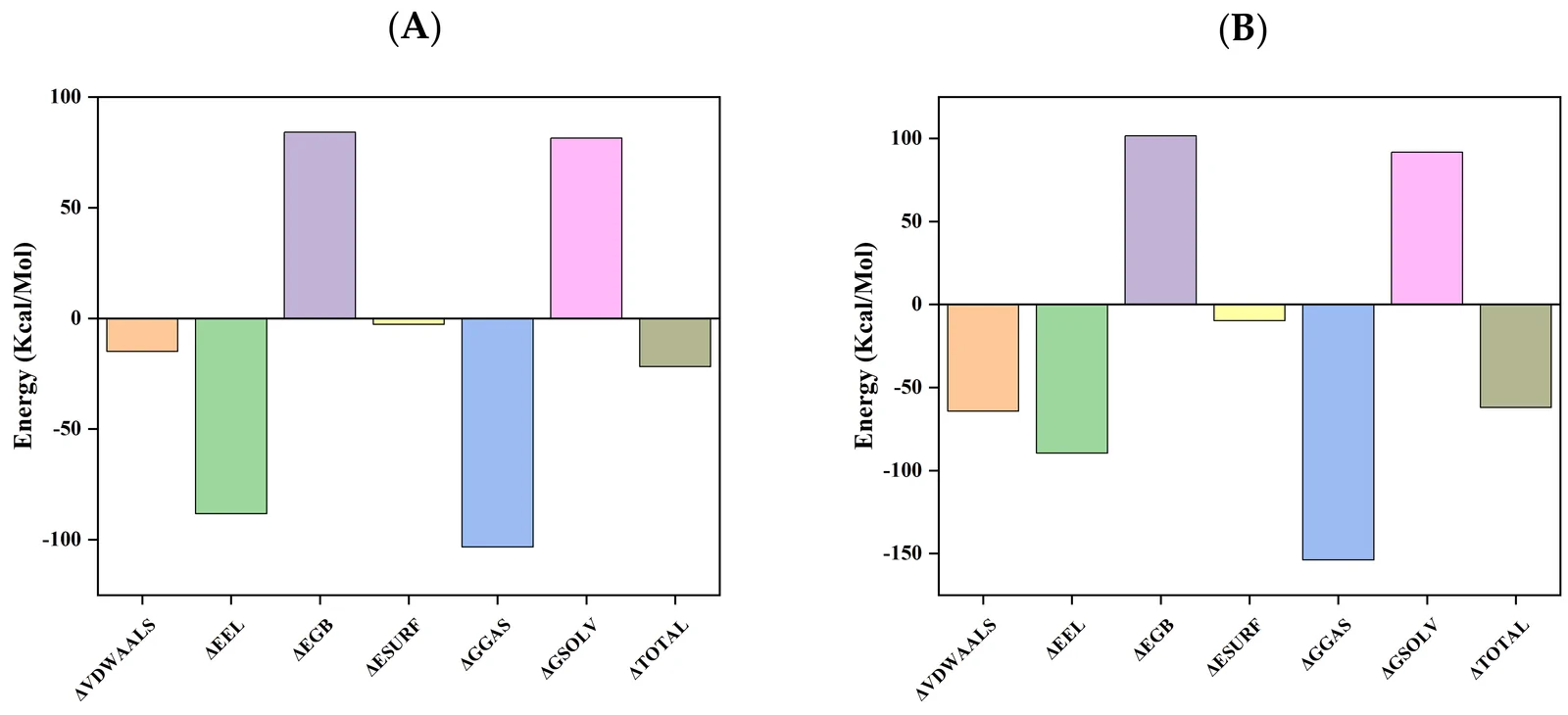

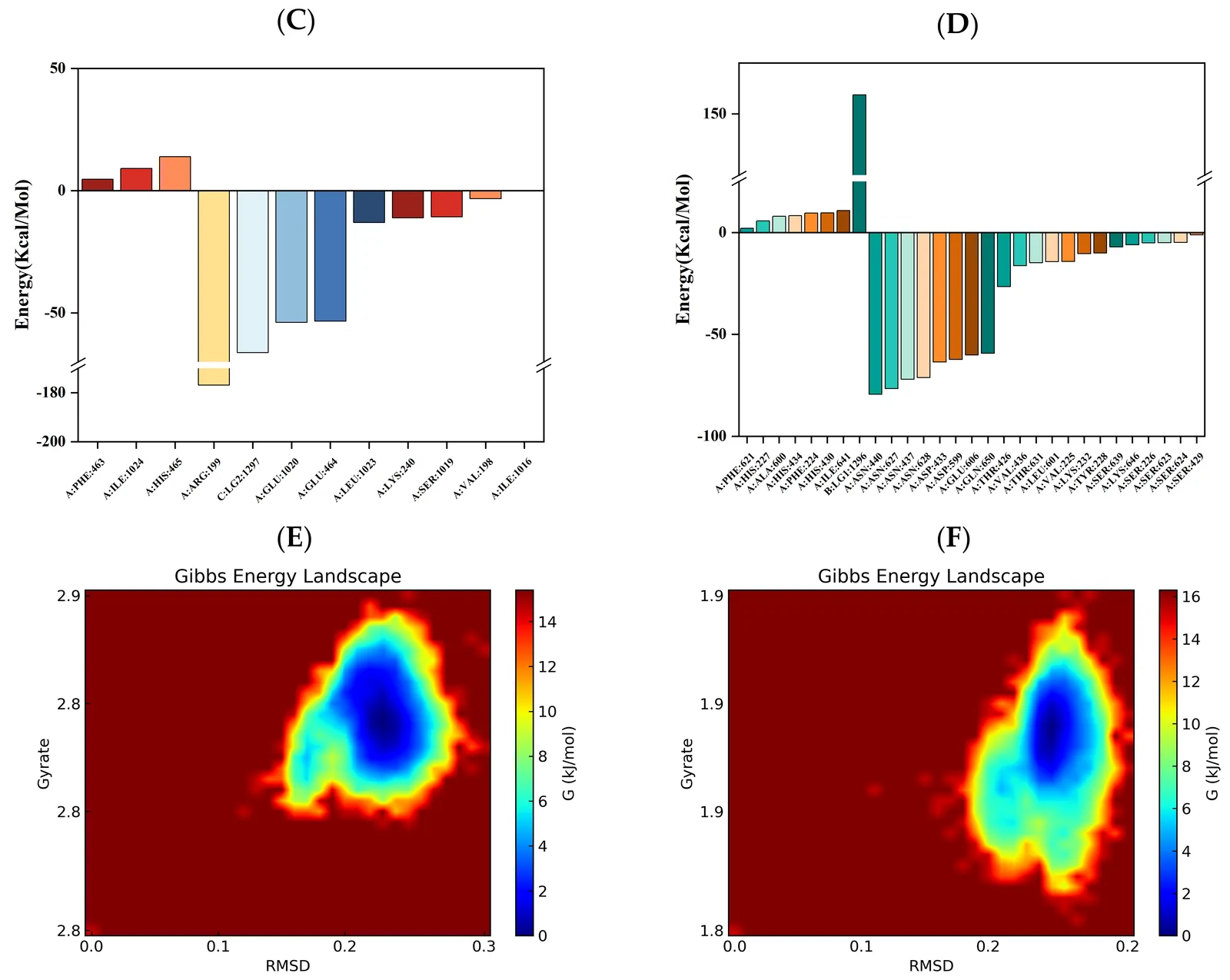
Binding-energy components for Lys (**A**) and KGM (**B**), calculated separately from the same Lys–KGM–myosin trajectory; per-residue energy contributions for Lys (**C**) and KGM (**D**); The free energy landscape map of Myosin (**E**) and Lys-KGM-Myosin complex (**F**). Note: VDWAALS: van der Waals energy, EEL: Electrostatic energy, EGB: Polar solvation energy, ESURF: Non-polar solvation energy, GGAS: Total gas phase free energy, GSOLV: Total solvation free energy, TOTAL: GSOLV + GGAS.

Binding free energy was analyzed to evaluate the interaction strength between KGM/Lys and myosin. As illustrated in Figure 8A,B, the total binding free energies of both Lys-myosin and KGM-myosin systems were below zero, indicating thermodynamically favorable interactions [40]. Among the energy components, ΔVDWAALS, ΔEEL, and ΔGGAS showed negative values, suggesting that van der Waals forces and electrostatic interactions contributed to complex stability, whereas positive ΔEGB and ΔGSOLV values indicated unfavorable polar solvation contributions [50,51]. The total binding free energy of the KGM-myosin system was more negative than that of the Lys-myosin system, indicating stronger predicted binding stability of KGM with myosin.

The energy contribution maps further showed different residue contribution patterns between Lys and KGM. For the Lys-myosin system, ARG199A, GLU1020A, and GLU464A made the main favorable energy contributions (Figure 8C). For the KGM-myosin system, ASN440A, ASN627A, ASN437A, ASN628A, ASP433A, ASP599A, GLU606A, and GLN650A were the main favorable residues (Figure 8D). These results indicated that Lys and KGM interacted with myosin through different binding regions and energy contribution patterns.

The free energy landscape was further used to describe conformational stability during simulation. As shown in Figure 8E,F, both systems showed low-energy basins, whereas the Lys-KGM-myosin complex exhibited a more concentrated low-energy region at a lower Rg range, suggesting that the complex tended to adopt a relatively stable and compact conformational state during simulation. Overall, the molecular dynamics results provided supporting evidence for the docking analysis, suggesting that Lys and KGM might interact with myosin through different molecular interaction modes and contribute to stabilization of the complex.

### 2.12. Proposed Hypothesis for the Improvement of Low-Salt MP Gels by Lys and KGM

**Figure 9:**
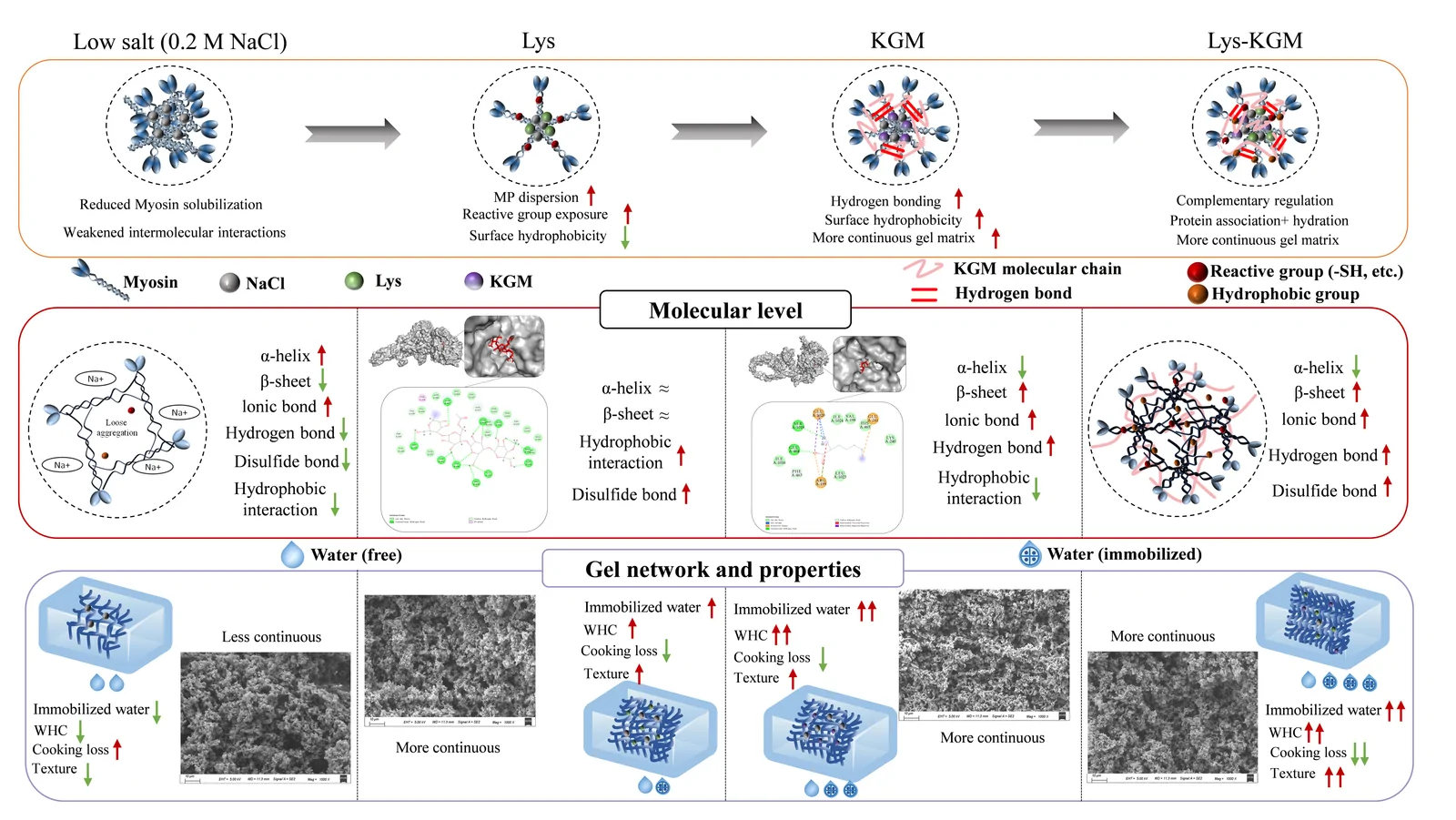
Schematic diagram of plausible pathways involved in low-salt MP gels regulation by Lys and KGM. Upward arrows (↑) represent an increase of corresponding indicators, while downward arrows (↓) represent a decrease.

Based on the overall experimental results, a hypothesis for the improvement of low-salt MP gels by Lys and KGM is proposed in Figure 9. Under low-salt conditions, insufficient ionic strength limited MP solubilization and unfolding, thereby weakening intermolecular association during heating. This led to a loose gel network with enlarged pores, allowing more water to migrate from immobilized water to mobile water and leading to higher cooking loss, lower WHC, weaker texture, and a less continuous microstructure. Lys and KGM might improve low-salt MP gel formation in different ways. Lys addition increased reactive sulfhydryl content and surface hydrophobicity, which might facilitate hydrophobic association and disulfide-bond formation during heating. KGM addition reduced surface hydrophobicity and enhanced water immobilization. These changes might be related to the strong hydration capacity of KGM and its possible non-covalent interactions with MP. These effects might help restrict water migration and maintain gel-network continuity. In the Lys-KGM group, modest changes in protein conformation and the relative contributions of intermolecular forces were accompanied by enhanced water immobilization and gel-network continuity. Docking and molecular dynamics simulations predicted different binding regions and interaction patterns for Lys and KGM with myosin. KGM showed a stronger predicted binding affinity than Lys, and its predicted interactions mainly involved hydrogen bonds, van der Waals forces, and carbon-hydrogen bonds. In contrast, the predicted Lys-myosin interaction was more strongly affected by charge-related interactions. These results supported the possibility of different interactions of Lys and KGM with myosin but did not directly demonstrate these interactions during experimental gel formation. Overall, the improved gel quality of the Lys-KGM group might be related to changes in protein conformation, intermolecular interactions, water state, and gel-network organization.

## 3. Conclusions

This study showed that the addition of Lys and/or KGM improved the properties of low-salt beef MP gels. Compared with the 0.2 M NaCl group, both Lys and KGM reduced cooking loss and improved WHC and texture. Among the low-salt treatments, the Lys-KGM group exhibited the lowest cooking loss, the highest WHC and immobilized-water content, and the most continuous gel network. Lys treatment increased reactive sulfhydryl content and BPB-binding capacity, whereas KGM treatment reduced BPB-binding capacity and enhanced water immobilization. The Lys-KGM group showed small but statistically significant changes in secondary-structure composition, together with changes in the relative contributions of intermolecular forces. Molecular docking and molecular dynamics (MD) simulations predicted different interaction patterns of Lys and the modeled KGM ligand with myosin, with the KGM ligand showing a stronger predicted binding affinity than Lys. Overall, the improved gel properties might be associated with changes in protein conformation, intermolecular interactions, water state, and gel-network organization. The results may provide a practical approach to improving the quality of reduced-sodium meat protein gels. However, because the present study was based on a beef MP model gel system, further studies using actual reduced-sodium meat formulations are needed to confirm the effects of Lys and KGM and to evaluate sodium content, sensory acceptability, saltiness perception, digestion behavior, and consumer acceptance.

## 4. Materials and Methods

### 4.1. Materials and Chemicals

Fresh Longissimus dorsi muscle samples were obtained from three individual cattle at Suguo Supermarket (Yangzhou, China). Lys and KGM were obtained from Aladdin Biochemical Technology Company (Shanghai, China). All chemicals were of analytical grade.

### 4.2. Extraction of MP

MP was extracted independently from each muscle sample. MP extraction was performed according to Xu et al. [52] with slight modifications. All operations were performed at 4 °C. Beef samples were homogenized with buffer at a ratio of 1:3 (*w*/*v*). Four cycles of homogenization (30 s) and centrifugation (2000× *g*, 10 min) were performed. 0.01 M phosphate-buffered saline (PBS) was used for the first two cycles, and 0.1 M NaCl buffer was used for the last two cycles. The precipitate was collected for use.

### 4.3. Sample Preparation

For each of the three independently prepared biological batches, the resulting MP pellet was divided into five portions, each of which was separately resuspended in one of the five solutions prepared in 20 mM PBS (pH 7.0): 0.6 M NaCl (high-salt control), 0.2 M NaCl (low-salt control), 0.2 M NaCl with 0.4% Lys (Lys), 0.2 M NaCl with 0.5% KGM (KGM), and 0.2 M NaCl with 0.4% Lys and 0.5% KGM (Lys-KGM). All Lys and KGM concentrations are expressed as w/v. The Lys and KGM concentrations were selected based on previous studies [12,17,18] and preliminary single-factor experiments evaluating cooking loss and gel hardness. The suspensions were then kept at 4 °C until use. For gel preparation, 5 g of MP suspension (40 mg/mL) was heated from 25 °C to 75 °C at 1 °C/min and held at 75 °C for 30 min, followed by cooling in an ice-water bath for 20 min.

### 4.4. Cooking Loss

The combined weight of the centrifuge tube and sample was measured before heating (*m*_1_) and after heating (*m*_2_). *Cooking loss* (%) was calculated as:

(1) $$Cooking\;loss\;\left(\%\right)=\;100\;\times \frac{\left(m_{1}-m_{2}\right)}{\left(m_{1}-m_{0}\right)}\;$$

where *m*_0_ refers to the weight of empty centrifuge tube.

### 4.5. WHC of Gels

Gels were blotted to remove surface moisture and centrifuged at a relative centrifugal force of 5000× *g* for 10 min at 4 °C. The weight of the empty tube (*M*_0_), tube with sample before centrifugation (*M*_1_), and tube with sample after centrifugation (*M*_2_) were recorded. *WHC* (%) was calculated as follows:

(2) $$WHC\;\left(\%\right)=100\times \frac{\left(M_{2}-M_{0}\right)}{{(M}_{1}-M_{0})}\;$$

### 4.6. Low-Field Nuclear Magnetic Resonance (LF-NMR) of MP Gels

Gel samples were cut into cylinders (diameter 1 cm) and analyzed using an AccuFat-1050 LF-NMR analyzer (Shanghai Niumag Electronic Technology Co., Ltd., Shanghai, China) with a Carr-Purcell-Meiboom-Gill (CPMG) pulse sequence. Each sample was scanned 16 times with a 2 s interval and a receiver gain of 4. The obtained CPMG decay signals were subjected to inverse Laplace transformation using the manufacturer’s software (version 4.02, Magmai, Nanjing, China). The transverse relaxation time (T2) distributions were calculated using the non-negative CONTIN algorithm with 200 logarithmically spaced points within the range of 0.1–10,000 ms. The individual relaxation components (T_2b_, T_21_, and T_22_) were identified based on the valleys between adjacent peaks in the averaged T2 distribution, and the corresponding peak areas were expressed as percentages of the total LF-NMR signal intensity.

### 4.7. Texture Analysis of MP Gels

Texture profile analysis (TPA) of the MP gels was performed using a TA.XT2i texture analyzer (Stable Micro Systems Ltd., Godalming, Surrey, UK) equipped with a 50-mm-diameter cylindrical probe (P/50). The gels were cut into cubes measuring 10 × 10 × 10 mm. Each sample was subjected to two compression cycles to 50% of its original height, with an interval of 5.0 s between cycles. The pre-test, test, and post-test speeds were 120, 60, and 60 mm/min, respectively, and the trigger force was 5 g. Neither the probe nor the sample surface was lubricated during testing.

### 4.8. Dynamic Rheological Measurements

The rheological properties of MP suspensions (10 mg/mL) were measured using a HAAKE MARS III rheometer (Thermo Electron GmbH, Karlsruhe, Germany). Measurements were conducted using a 35 mm parallel plate with a 1 mm gap. The linear viscoelastic region was determined at 25 °C by a strain-amplitude sweep from 0.01% to 100% at a constant frequency of 1 Hz. Its upper limit was defined as the strain at which G′ decreased by more than 5% from its plateau value. A strain of 1%, which was within the linear viscoelastic region for all treatments, was used for the subsequent oscillatory measurements. Silicone oil was applied around the edge of the sample to minimize moisture loss during testing.

For the frequency sweep, G′ and G″ were recorded over a frequency range of 0.1–16 Hz at 25 °C and 1% strain. The apparent viscosity was measured at 25 °C over a shear-rate range of 0.1–100 s^−1^. The resulting apparent-viscosity data were fitted using the Power-Law model:

(3) $$\eta =K\gamma^{n-1}$$

where *η* represents the apparent viscosity (Pa·s), *γ* represents the shear rate (s^−1^), *K* represents the consistency coefficient (Pa·s^n^), and *n* represents the flow behavior index. The parameters *K* and *n* were obtained by nonlinear regression, and the coefficient of determination (R^2^) was used to evaluate the goodness of fit. Values of *n* < 1 indicate shear-thinning behavior.

For the temperature sweep, G′, G″, and tan δ were continuously recorded while the temperature was increased from 20 to 80 °C at a heating rate of 1 °C/min, a frequency of 1 Hz, and a strain of 1%. Three independently prepared MP suspensions were analyzed for each treatment.

### 4.9. Scanning Electron Microscopy (SEM)

Samples (2 mm × 2 mm × 1 mm) were immersed in 2.5% glutaraldehyde at 4 °C for 12 h. Then, the samples were rinsed thrice with 0.1 M PBS (pH 7.2) and dehydrated stepwise in 50%, 70%, 80%, 90%, and 100% ethanol for 15 min at each concentration. After freeze-drying, the samples were gold-coated and observed using a GeminiSEM 300 scanning electron microscope (Carl Zeiss Microscopy GmbH, Jena, Germany) at 5 kV and a magnification of 1000×. A specimen from the central region of each gel was examined, and three randomly selected non-overlapping fields were captured. Representative micrographs were selected from the acquired images according to the typical pore structure and network characteristics of each treatment.

### 4.10. Total and Reactive Sulfhydryl Groups

Sulfhydryl content was determined following Guo et al. [40] with modifications. Total sulfhydryl (T-SH) content was measured by mixing 1 mL of MP solution (5 mg/mL) with 2 mL of Tris-glycine buffer containing 10.4 mg/mL Tris, 6.9 mg/mL glycine, 1.2 mg/mL EDTA, and 480 mg/mL urea (pH 8.0). Then, 0.02 mL of Ellman’s reagent was added, and the mixture was incubated at 25 °C for 5 min in the dark. Absorbance was recorded at 412 nm.

Reactive sulfhydryl (R-SH) content was measured in the same way, except that urea was removed from the buffer. The sulfhydryl contents were expressed as μmol/g protein using a molar extinction coefficient of 13,600 M^−1^ cm^−1^.

### 4.11. Surface Hydrophobicity of MP

Surface hydrophobicity was measured using bromophenol blue (BPB) binding [53]. 1 mL of MP suspension (5 mg/mL) was mixed with 0.2 mL BPB solution (1 mg/mL), vortexed for 10 min, and centrifuged at 4000× *g* for 15 min at 4 °C. The supernatant was diluted 10-fold with PBS before measuring absorbance at 595 nm. Surface hydrophobicity was determined as the amount of BPB bound per mg protein using the following equation.

(4) $$H_{BPB}\left(\mathrm{\mu g}\;\mathrm{BPB}/\mathrm{mg}\;\mathrm{protein}\right)=\frac{200\;\mathrm{\mu g}\times (A_{0}-A_{1})/A_{0}}{5}$$

where *A*_0_ is the absorbance of the BPB solution without protein, and *A*_1_ is the absorbance of the corresponding sample supernatant.

### 4.12. Fourier Transform Infrared Spectroscopy (FT-IR)

Lyophilized gel samples were mixed with dried KBr at a mass ratio of 1:50 (*w*/*w*), ground thoroughly, and compressed into 1-mm-thick pellets. FTIR spectra were collected using a Cary 610/670 FTIR spectrometer (Varian, Inc., Palo Alto, CA, USA) over the range of 4000–400 cm^−1^ at a spectral resolution of 4 cm^−1^, with 32 scans per spectrum. A KBr background spectrum was recorded under the same conditions and used for background correction. Three independently prepared gel samples were analyzed for each treatment. Raw spectra were subjected to multipoint baseline correction and min–max normalization using the instrument software. The amide I region (1600–1700 cm^−1^) was analyzed using PeakFit v4.12 (SeaSolve Software Inc., Framingham, MA, USA). Peak positions were identified from the second-derivative spectra after nine-point Savitzky–Golay smoothing. Overlapping bands were fitted with Gaussian functions using nonlinear least-squares analysis, with the peak positions constrained around those identified from the second-derivative spectra. The fitting was accepted when the coefficient of determination was ≥0.99 and the residual curve showed no systematic pattern. Following Gao et al. [18], the fitted peaks were assigned to β-sheet (1600–1640 and 1670–1690 cm^−1^), random coil (1640–1650 cm^−1^), α-helix (1650–1658 cm^−1^), and β-turn (1660–1700 cm^−1^). The relative content of each secondary-structure component was calculated by dividing the summed areas of its assigned fitted peaks by the total area of all fitted peaks in the amide I region and multiplying by 100%.

### 4.13. Determination of the Intermolecular Interactions

Intermolecular forces in MP gels were determined according to Xu et al. [25] with minor modifications. 2 g of gel samples was homogenized with 18 mL of five extraction solutions: SA, 0.05 M NaCl; SB, 0.6 M NaCl; SC, SB with 1.5 M urea; SD, SB with 8 M urea; and SE, SB with 8 M urea and 0.05 M β-mercaptoethanol. The mixtures were heated at 80 °C for 30 min and then cooled to 25 °C. The supernatant was collected for protein quantification after centrifugation at 5000× *g* for 15 min. Soluble protein was quantified by the Biuret method using bovine serum albumin standards (0–10 mg/mL), and absorbance was measured at 540 nm against a reagent blank. Differences in solubility were used to estimate ionic bonds, hydrophobic interactions, hydrogen bonds, and disulfide bonds. The intermolecular forces were defined using the following equations:

(5) $$Ionic\;bonds=C_{SB}-C_{SA}$$

(6) $$Hydrogen\;bonds=C_{SC}-C_{SB}$$

(7) $$Hydrophobic\;interactions=C_{SD}-C_{SC}$$

(8) $$Disulfide\;bonds=C_{SE}-C_{SD}$$

where *C_SA_*, *C_SB_*, *C_SC_*, *C_SD_*, and *C_SE_* represent the soluble protein concentrations obtained from extraction solutions SA, SB, SC, SD, and SE, respectively.

### 4.14. Molecular Docking

The full-length AlphaFold-predicted structure of bovine myosin-VI was used as the receptor (MYO6; UniProtKB accession E1BPK6; model AF-E1BPK6-F1, version 6). The model was used in its full-length form as downloaded, without residue truncation or missing-residue reconstruction. Polar hydrogen atoms and Gasteiger charges were added using AutoDockTools 1.5.6, and the receptor was saved in PDBQT format. The three-dimensional structure of L-lysine was obtained from PubChem (CID 5962). Because commercial KGM is a heterogeneous high-molecular-weight polysaccharide, a glucomannan tetrasaccharide obtained from PubChem (CID 24892726; DP = 4) was used as a simplified model of its local chain structure, following Gao et al. [1]. At pH 7.0, Lys was modeled in its zwitterionic form with a net charge of +1, whereas the glucomannan tetrasaccharide was neutral.

Lys and the glucomannan tetrasaccharide were docked separately to full-length MYO6 model using AutoDock Vina 1.1.2. Blind docking was performed using a grid centered at (x, y, z) = (14.837, −3.117, −6.758) Å, with dimensions of 87 × 93 × 115 Å. The exhaustiveness value was set to 32, and 10 poses were generated for each ligand. The pose with the lowest predicted binding energy was retained for subsequent analysis. Protein–ligand interactions were visualized using Discovery Studio 2019 Client.

### 4.15. Molecular Dynamics Simulation

MD simulations were performed using GROMACS 2022.3 [54] for unbound full-length MYO6 and a ternary Lys–glucomannan-tetrasaccharide–MYO6 complex constructed from the selected docking poses. MYO6 was described using AMBER99SB-ILDN, whereas the ligands were parameterized using GAFF with RESP charges calculated at the HF/6-31G(d) level using Gaussian 16W and AmberTools 22. Each system was placed in an orthorhombic box with a minimum solute-to-boundary distance of 1.0 nm, solvated using TIP3P water, and neutralized with counterions; no additional NaCl was added.

Energy minimization was performed by steepest descent for up to 50,000 steps or until the maximum force was below 1000 kJ mol^−1^ nm^−1^. The systems were equilibrated for 100 ps under NVT conditions at 300 K and for 100 ps under NPT conditions at 300 K and 1 bar. Temperature was controlled using the velocity-rescaling thermostat (τ = 0.1 ps), and pressure was controlled using the Parrinello–Rahman barostat (τ = 2.0 ps). A 100-ns production simulation was performed using a 2-fs time step. Short-range electrostatic and van der Waals cutoffs were 1.0 nm; long-range electrostatics were treated using PME, and bonds involving hydrogen atoms were constrained using LINCS. One independent trajectory was generated per system and analyzed for RMSD, RMSF, Rg, SASA, and hydrogen bonds.

Ligand-specific binding energies were calculated separately from the ternary-complex trajectory using gmx_MMPBSA v1.5.6 and MM/GBSA. Ten snapshots were extracted from 91–100 ns at 1-ns intervals. Calculations used the GB-OBC2 model (igb = 5), internal/external dielectric constants of 1.0/80.0, an ionic strength of 0.0 M, the LCPO nonpolar-solvation model (gbsa = 2), and pairwise residue decomposition (idecomp = 2). Entropy was not included; therefore, the calculated energies were used for relative comparison. Because KGM was represented by a short oligosaccharide and only one trajectory was generated per system, the computational results were interpreted as evidence of possible local interactions with the full-length MYO6 model rather than direct proof of interactions during experimental gel formation.

### 4.16. Statistical Analysis

Data were presented as mean ± standard deviation (SD) from three independent biological replicates (*n* = 3). The main effects of Lys and KGM and their interaction in the four low-salt treatments were evaluated using two-way analysis of variance (ANOVA). Differences were considered statistically significant at *p* < 0.05. Statistical analyses were performed using SPSS 26.0 (IBM Corp., Armonk, NY, USA). Figures were plotted with GraphPad Prism 10 (GraphPad Software Inc., Boston, MA, USA), and Origin 9.0 software (OriginLab Corporation, Northampton, MA, USA).

## Figures and Tables

**Table 1:** Texture properties of MP gels under different treatments.

Group	Hardness (N)	Springiness (mm)	Cohesiveness (Ratio)	Adhesion (mJ)	Gumminess (N)	Chewiness (mJ)
0.6 M NaCl	2.80 ± 0.17 ^b^	1.20 ± 0.15 ^a^	0.24 ± 0.03 ^ab^	0.26 ± 0.09 ^c^	0.71 ± 0.11 ^a^	0.84 ± 0.04 ^a^
0.2 M NaCl	1.83 ± 0.06 ^d^	0.46 ± 0.02 ^d^	0.18 ± 0.01 ^b^	0.89 ± 0.11 ^a^	0.56 ± 0.05 ^b^	0.31 ± 0.04 ^d^
Lys	2.20 ± 0.10 ^c^	0.58 ± 0.01 ^cd^	0.26 ± 0.02 ^a^	0.53 ± 0.08 ^b^	0.54 ± 0.07 ^b^	0.31 ± 0.05 ^d^
KGM	2.87 ± 0.12 ^b^	0.70 ± 0.08 ^bc^	0.28 ± 0.04 ^a^	0.66 ± 0.19 ^ab^	0.80 ± 0.11 ^a^	0.56 ± 0.08 ^c^
Lys-KGM	3.10 ± 0.10 ^a^	0.83 ± 0.04 ^b^	0.28 ± 0.01 ^a^	0.78 ± 0.17 ^a^	0.86 ± 0.04 ^a^	0.71 ± 0.05 ^b^

Note: The values are the mean ± standard deviation. Different lowercase letters in the same column indicate statistically significant differences (*p* < 0.05) (*n* = 3).

**Table 2:** Power-law model parameters of different samples.

Group	*K* (Pa·s^n^)	*n*	R^2^
0.6 M NaCl	6.628	0.177	0.981
0.2 M NaCl	12.130	0.060	0.986
Lys	10.283	0.118	0.993
KGM	21.536	0.221	0.999
Lys-KGM	32.379	0.147	0.999

Note: *K*, consistency coefficient; *n*, flow behavior index; R^2^, coefficient of determination.

**Table 3:** Sulfhydryl content and surface hydrophobicity of MP under different treatments.

Group	R-SH (μmol/g)	T-SH (μmol/g)	Surface Hydrophobicity (μg BPB/mg Protein)
0.6 M NaCl	1.81 ± 0.04 ^a^	2.36 ± 0.03 ^a^	21.90 ± 0.22 ^a^
0.2 M NaCl	1.59 ± 0.02 ^c^	2.34 ± 0.04 ^a^	18.30 ± 0.40 ^c^
Lys	1.80 ± 0.01 ^a^	2.32 ± 0.02 ^a^	19.17 ± 0.18 ^b^
KGM	1.73 ± 0.04 ^b^	2.38 ± 0.11 ^a^	10.97 ± 0.10 ^e^
Lys-KGM	1.58 ± 0.01 ^c^	2.35 ± 0.13 ^a^	14.37 ± 0.40 ^d^

Note: The values are the mean ± standard deviation. Different lowercase letters in the same column indicate statistically significant differences (*p* < 0.05) (*n* = 3).

## Data Availability

The data presented in this study are available on request from the corresponding authors. The data are not publicly available because they are part of ongoing research.
